# Supplementary figures and images for: A wake-active locomotion circuit depolarizes a sleep-active neuron to switch on sleep
Source: PLoS Biol. 2020 Feb 20;18(2):e3000361. doi: 10.1371/journal.pbio.3000361 (PMC7053779; doi:10.1371/journal.pbio.3000361)

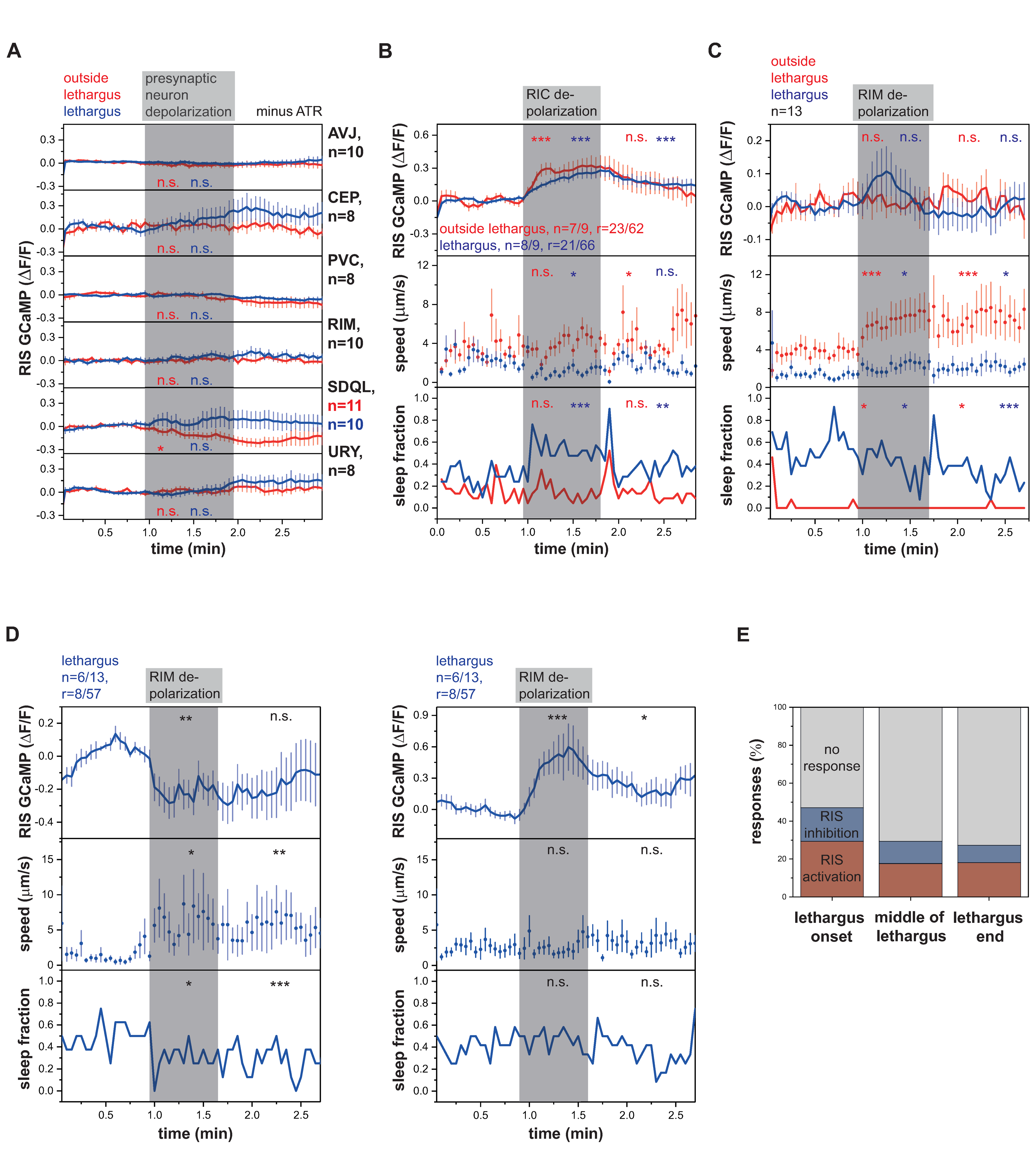

Supplement: S1 Fig — (A) Control experiments. Optogenetic depolarization of RIS presynaptic neurons without the addition of ATR. For statistical calculations, baseline neural activities (0–0.95 min) were compared to neural activity levels during the stimulation period (1–1.95 min). *p < 0.05, **p < 0.01, Wilcoxon signed rank test for GCaMP (S2 Data, Sheet S1A). (B) Optogenetic RIC depolarization induced an RIS activity increase outside of and during lethargus. An average of all responsive trials is shown in this figure. Trials were classified as responsive or nonresponsive. In responsive trials, an RIS activity increase correlated with the onset of the stimulation period. In nonresponsive trials, no change in RIS activity levels could be seen. “n” represents the number of animals tested, and “r” represents the number of trials. For statistical analysis, RIS baseline activity levels (0–0.95 min) were compared to activity levels during (1–1.95 min) and after (2–2.95 min) the stimulation. *p < 0.05, **p < 0.01, ***p < 0.001, Wilcoxon signed rank test for GCaMP and speed, Fisher’s exact test for sleep fraction (S2 Data, Sheet S1B). (C) Depolarization of RIM using ReaChR expressed under the gcy-13 promoter had no net effect on RIS function. Neural baseline activity levels (0–0.95 min) were compared to neuronal levels during the stimulation (1–1.95 min) and after the stimulation (2–2.95 min). *p < 0.05, **p < 0.01, ***p < 0.001, Wilcoxon signed rank test for GCaMP and speed, Fisher’s exact test for sleep fraction (S2 Data, Sheet S1C-E). (D) RIM optogenetic depolarization using ReaChR expressed under the gcy-13 promoter induced either RIS activation or inhibition. Single trials were classified as activating if an activity increase in RIS correlated with onsets of optogenetic stimulation periods. Trials were classified as inhibitory if an activity decrease in RIS correlated with onsets of optogenetic stimulation periods. “n” represents the number of animals tested, and “r” represents the number [file pbio.3000361.s001.tif]

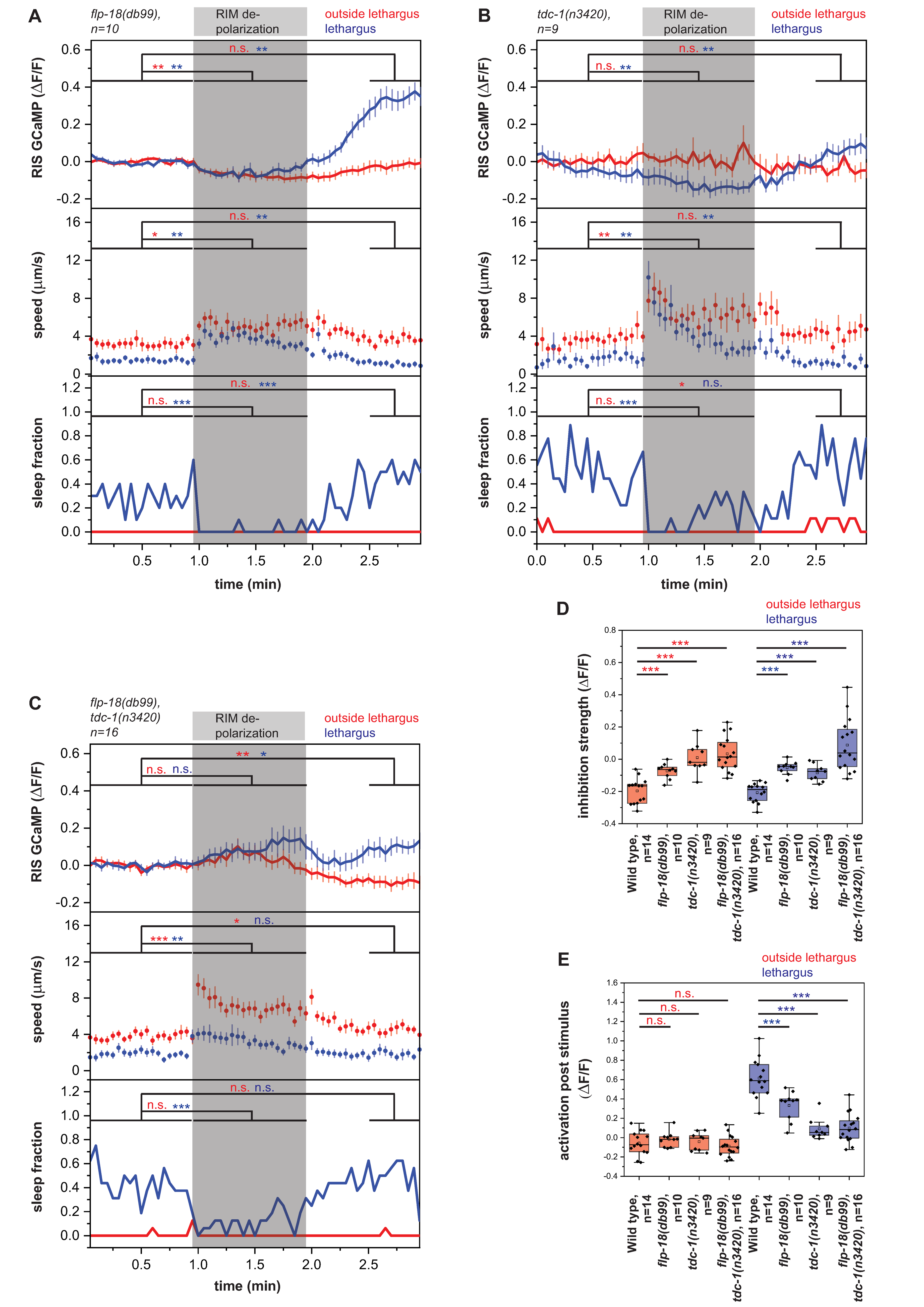

Supplement: S2 Fig — Optogenetic RIM manipulations in these experiments were all performed with ReaChR expressed from the tdc-1 promoter. (A) Optogenetic RIM depolarization in flp-18(db99) single mutants. Outside of lethargus, RIS inactivation caused by RIM optogenetic depolarization was reduced to 37% of wild-type inhibition levels. During lethargus in flp-18(db99) mutants, animal inhibition levels were only 25% of wild-type level. Neuronal activity levels before (0–0.95 min), during (1–1.95 min), and after (2.5–2.95 min) optogenetic RIM depolarization were compared. *p < 0.05, **p < 0.01, ***p < 0.001, Wilcoxon signed rank test for GCaMP and speed, Fisher’s exact test for sleep fraction (S2 Data, Sheet S2A). (B) Optogenetic RIM depolarization in tdc-1(n3420) single mutants. Outside of lethargus, optogenetic RIM depolarization in tdc-1(n3420) single mutants no longer induced changes in RIS activity levels. During lethargus, inhibition levels during the stimulation period only reached 40% of wild-type levels. Neuronal activity levels before (0–0.95 min), during (1–1.95 min), and after (2.5–2.95 min) optogenetic RIM depolarization were compared. *p < 0.05, **p < 0.01, ***p < 0.001, Wilcoxon signed rank test for GCaMP and speed, Fisher’s exact test for sleep fraction (S2 Data, Sheet S2B). (C) Optogenetic RIM depolarization in flp-18(db99) and tdc-1(n3420) double mutants had no effect on RIS function. Neuronal activity levels before (0–0.95 min), during (1–1.95 min), and after (2.5–2.95 min) optogenetic RIM depolarization were compared. *p < 0.05, **p < 0.01, ***p < 0.001, Wilcoxon signed rank test for GCaMP and speed, Fisher’s exact test for sleep fraction (S2 Data, Sheet S2C). (D) Quantification of inhibition strength. RIS activity levels during optogenetic RIM depolarization in flp-18(db99), tdc-1(n3420) and flp-18(db99), and tdc-1(n3420) double mutants were compared to wild-type levels. Wild-type data are depicted in Fig 1B, RIM panel. Inhibition strength was calculated by subtracting [file pbio.3000361.s002.tif]

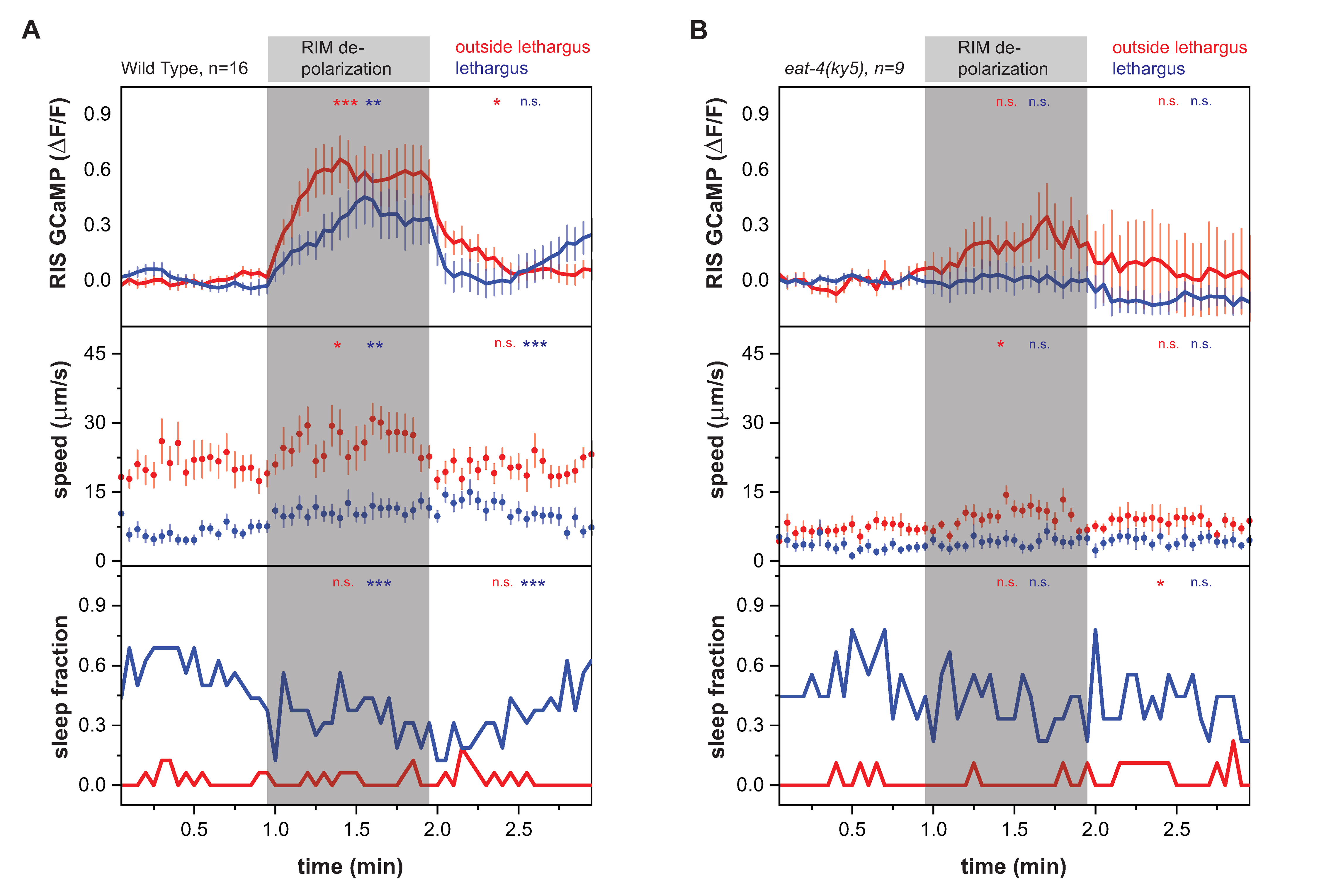

Supplement: S3 Fig — (A) RIM optogenetic depolarization using ReaChR expressed under the gcy-13 promoter induced robust RIS activation in L4 larvae. In the L4 larvae, RIS activation by RIM optogenetic depolarization was more robust compared with the same experiment in L1 larvae. No trial selection was required. For statistical analysis, RIS baseline activity levels (0–0.95 min) were compared to activity levels during (1–1.95 min) and after (2–2.95 min) the stimulation. *p < 0.05, **p < 0.01, ***p < 0.001, Wilcoxon signed rank test for GCaMP and speed, Fisher’s exact test for sleep fraction (S2 Data, Sheet S3A). (B) The activating input of RIM optogenetic depolarization on RIS was almost completely abolished in eat-4(ky5) mutants. For statistical analysis, RIS baseline activity levels (0–0.95 min) were compared to activity levels during (1–1.95 min) and after (2–2.95 min) the stimulation. *p < 0.05, **p < 0.01, ***p < 0.001, Wilcoxon signed rank test for GCaMP and speed, Fisher’s exact test for sleep fraction (S2 Data, Sheet S3B). (TIF) [file pbio.3000361.s003.tif]

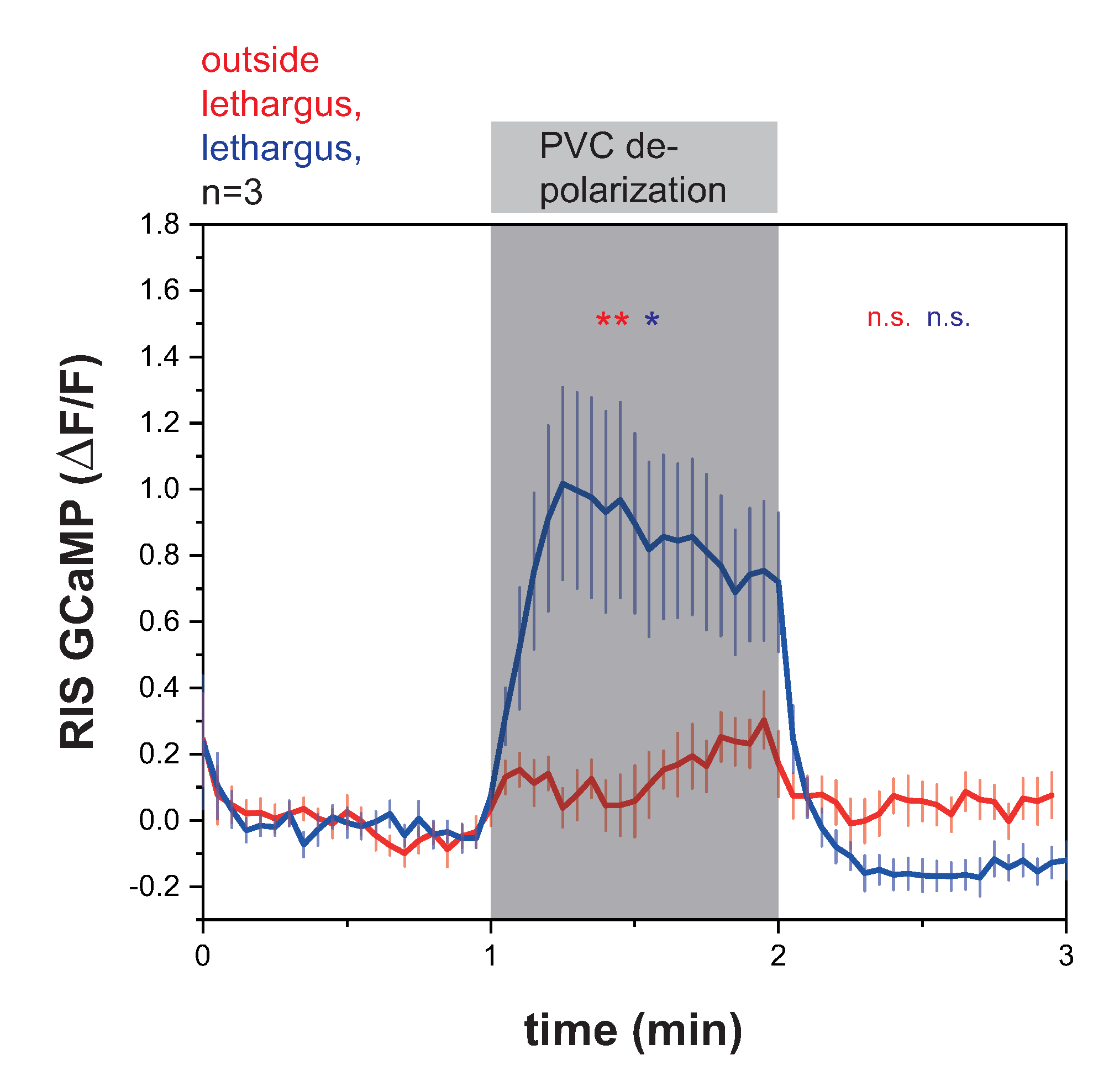

Supplement: S4 Fig — Optogenetic PVC depolarization in L2 larvae led to RIS activation outside of and during lethargus. RIS activation during lethargus was strongly enhanced. Plotted data represent the average over all experimental trials. Neural activity levels before the stimulation (0–0.95 min) were compared to activity levels during the stimulation (1–1.95 min). *p < 0.05, **p < 0.01, Wilcoxon signed rank test (S2 Data, Sheet S4). (TIF) [file pbio.3000361.s004.tif]

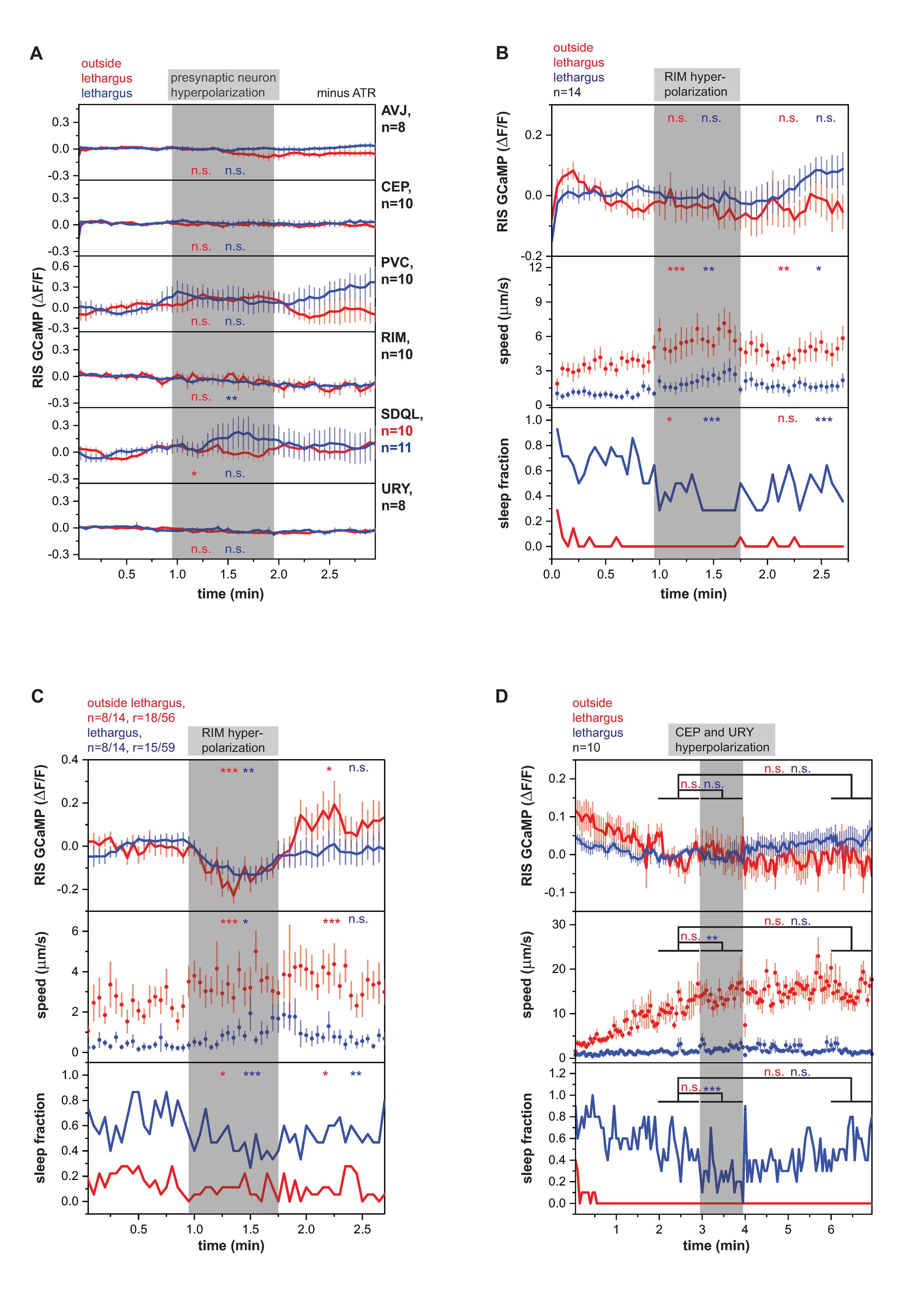

Supplement: S5 Fig — (A) Control experiments. Optogenetic hyperpolarization of RIS presynaptic neurons without the addition of ATR. For statistical calculations, baseline neural activities (0–0.95 min) were compared to neural activity levels during the stimulation period (1–1.95 min). *p < 0.05, **p < 0.01, Wilcoxon signed rank test for GCaMP (S2 Data, Sheet S5A). (B) Hyperpolarization of RIM using ArchT expressed under the gcy-13 promoter had no net effect on RIS function. Neural baseline activity levels (0–0.95 min) were compared to neuronal levels during the stimulation (1–1.95 min) and after the stimulation (2–2.95 min). *p < 0.05, **p < 0.01, ***p < 0.001, Wilcoxon signed rank test for GCaMP and speed, Fisher’s exact test for sleep fraction (S2 Data, Sheet S5B, C). (C) RIM optogenetic hyperpolarization using ArchT expressed under the gcy-13 promoter caused a decrease in RIS activity levels in selected trials. Single trials were classified as activating if an activity increase in RIS occurred at the onset of the optogenetic stimulation period. Trials were classified as inhibitory if an activity decrease in RIS occurred at the onset of the optogenetic stimulation period. “n” represents the number of animals tested, and “r” represents the number of trials. For statistical calculations, neural baseline activity levels (0–0.95 min) were compared to levels during the stimulation period (1–1.75 min). *p < 0.05, **p < 0.01, ***p < 0.001, Wilcoxon signed rank test for GCaMP and speed, Fisher’s exact test for sleep fraction (S2 Data, Sheet S5B, C). (D) Simultaneous optogenetic hyperpolarization of CEP and URY neurons does not induce changes in RIS activity levels. For statistical testing, baseline neural activities (2–2.95 min) were compared to neural activity levels during the stimulation period (3–3.95 min) and after the stimulation (6–6.95 min). **p < 0.01, ***p < 0.001, Wilcoxon signed rank test for GCaMP and speed, Fisher’s exact test for sleep fraction (S2 Data, Sheet S5D). (TIF) [file pbio.3000361.s005.tif]

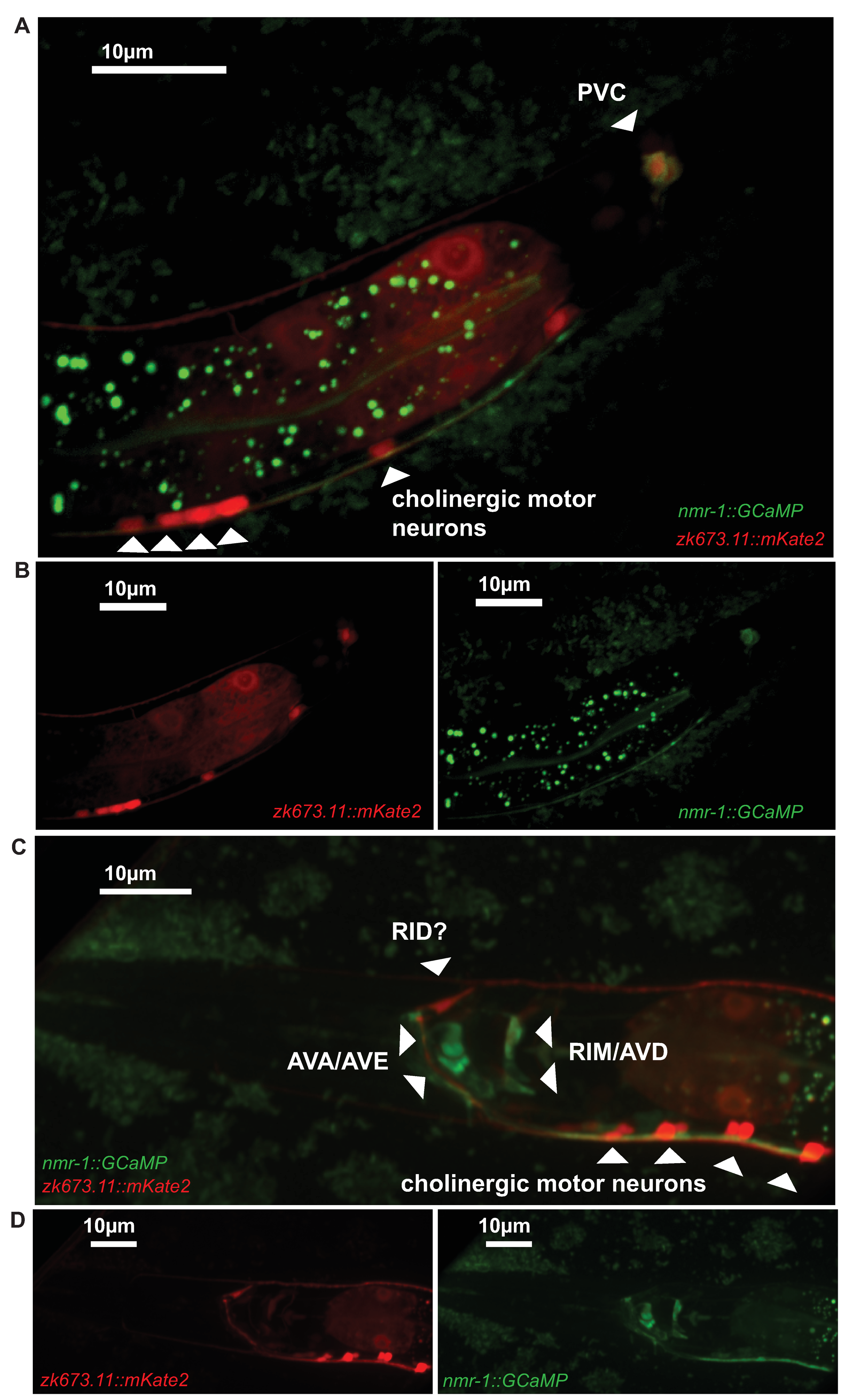

Supplement: S6 Fig — (A–B) Expression of nmr-1 and zk673.11 only overlaps in PVC in the tail. (C–D) Expression of nmr-1 and zk673.11 does not overlap in head neurons. (TIF) [file pbio.3000361.s006.tif]

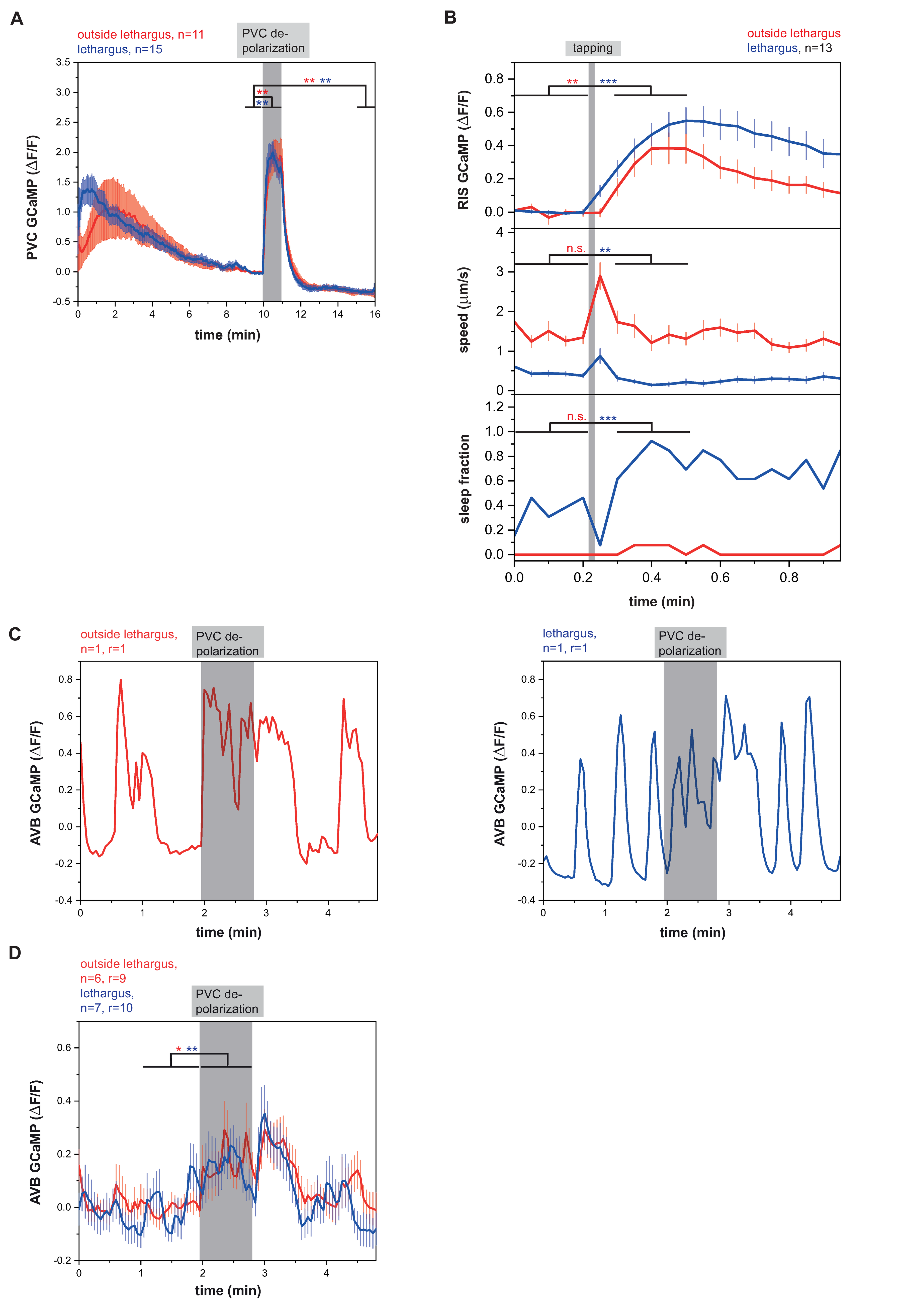

Supplement: S7 Fig — (A) PVC excitability remained unchanged during lethargus. Experiments were performed in immobilized L1 larvae to ensure PVC-specific green light illumination. A long baseline of 10 min was used to achieve stable baseline conditions. Activity levels of PVC during optogenetic depolarization were indistinguishable outside and during lethargus. PVC displayed a negative rebound transient after optogenetic depolarization. However, there was no difference in the amount of negative rebound outside and during lethargus (S2 Data, Sheet S7A). (B) RIS showed a rebound after mechanical stimulation. This rebound was stronger in worms during lethargus, and only during lethargus was the RIS rebound accompanied by a strongly increased immobilization of worms. **p < 0.01, ***p < 0.001, Wilcoxon signed rank test for GCaMP and speed, Fisher’s exact test for sleep fraction (S2 Data, Sheet S7B). (C–D) Effects of PVC stimulation on AVB activity. L1 larvae were immobilized for optogenetic experiments to ensure cell-specific stimulation of PVC. AVB activated upon optogenetic PVC depolarization with the same response strength during and outside of lethargus. AVB displayed an oscillatory activity pattern in 44% of all trials in worms outside of lethargus. AVB activity oscillated in 70% of all trials during lethargus. *p < 0.05, **p < 0.01, Wilcoxon signed rank test for GCaMP (S2 Data, Sheet S7C-D). (TIF) [file pbio.3000361.s007.tif]

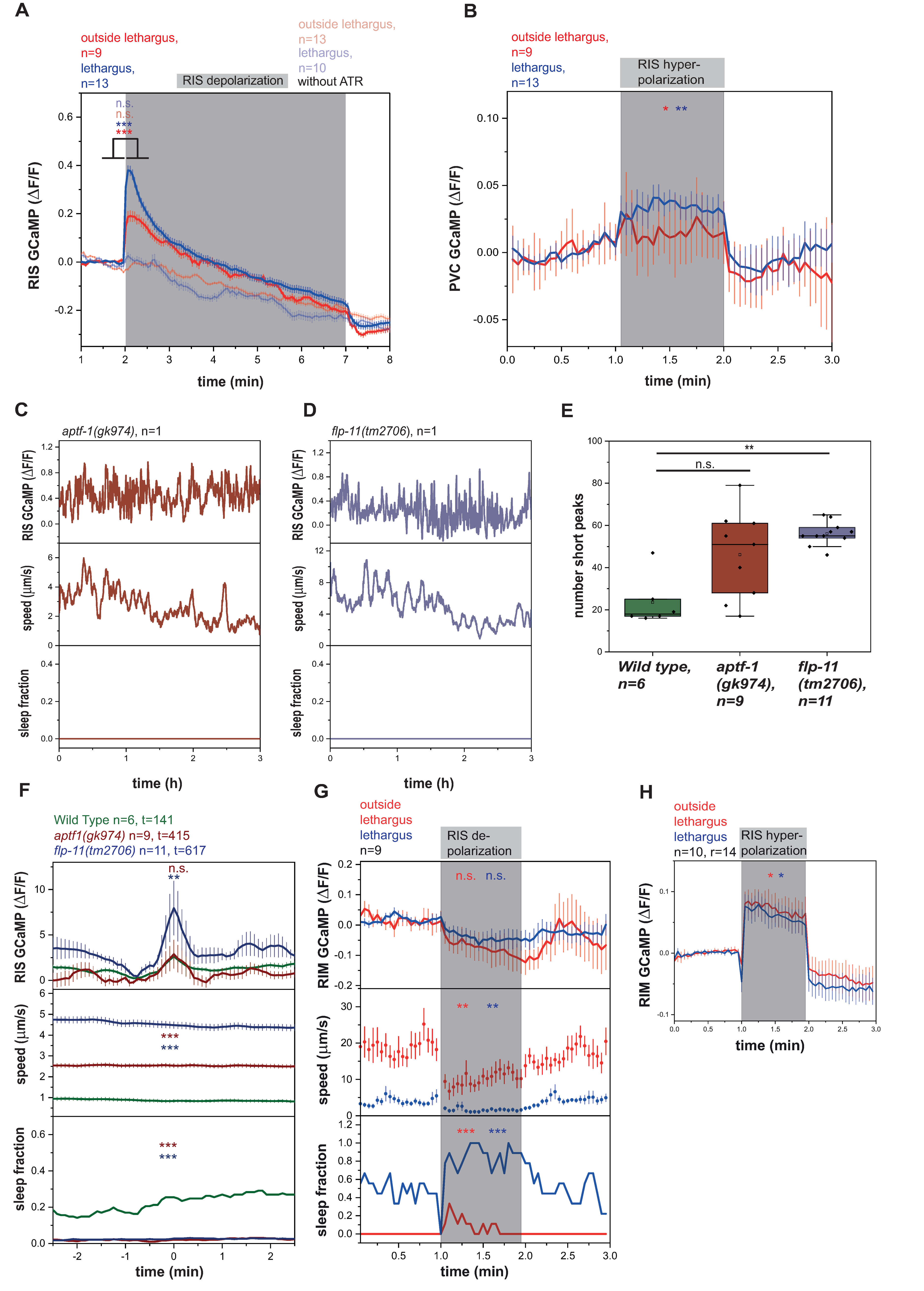

Supplement: S8 Fig — (A) RIS depolarizes during optogenetic activation in fixed animals. As controls, experiments were performed in the absence of ATR. ***p < 0.001, Wilcoxon signed rank test (S2 Data, Sheet S8A). (B) RIS hyperpolarization led to a weak PVC depolarization outside and during lethargus. For statistical calculations, neural activities before the stimulation period (0–1 min) were compared to activity levels during the stimulation period (1–2 min). *p < 0.05, **p < 0.01, compared before and during stimulation, Wilcoxon signed rank test (S2 Data, Sheet S8B). (C–D) Sample trace of RIS activity and worm locomotion behavior 3 h before shedding of the cuticle of aptf-1(gk794) and flp-11(tm2706) mutants (S2 Data, Sheet S8C and S8D). (E–F) flp-11(tm2706) mutants have a significantly increased number of short RIS peaks that do not correlate with sleep. (E) **p < 0.01, ***p < 0.001, Welch test. (F) **p < 0.01, Kolmogorov-Smirnov test (S1 Data, Sheet 3G-I). (G) Optogenetic RIS depolarization has no effect on RIM activity outside of and during lethargus. Neuronal activity levels before (0–0.95 min) and during (1–1.95 min) the stimulation period were compared. *p < 0.05, **p < 0.01, ***p < 0.001, Wilcoxon signed rank test for GCaMP and speed, Fisher’s exact test for sleep fraction (S2 Data, Sheet S8G). (H) Optogenetic RIS hyperpolarization induced increased RIM activity both outside of and during lethargus. Measurements were performed in immobilized L1 larvae to reduce measurement noise. Activity levels during baseline measurements (0–0.95 min) were compared to levels during optogenetic RIS manipulation (1–1.95 min). *p < 0.05, Wilcoxon signed rank test for GCaMP (S2 Data, Sheet S8H). (TIF) [file pbio.3000361.s008.tif]

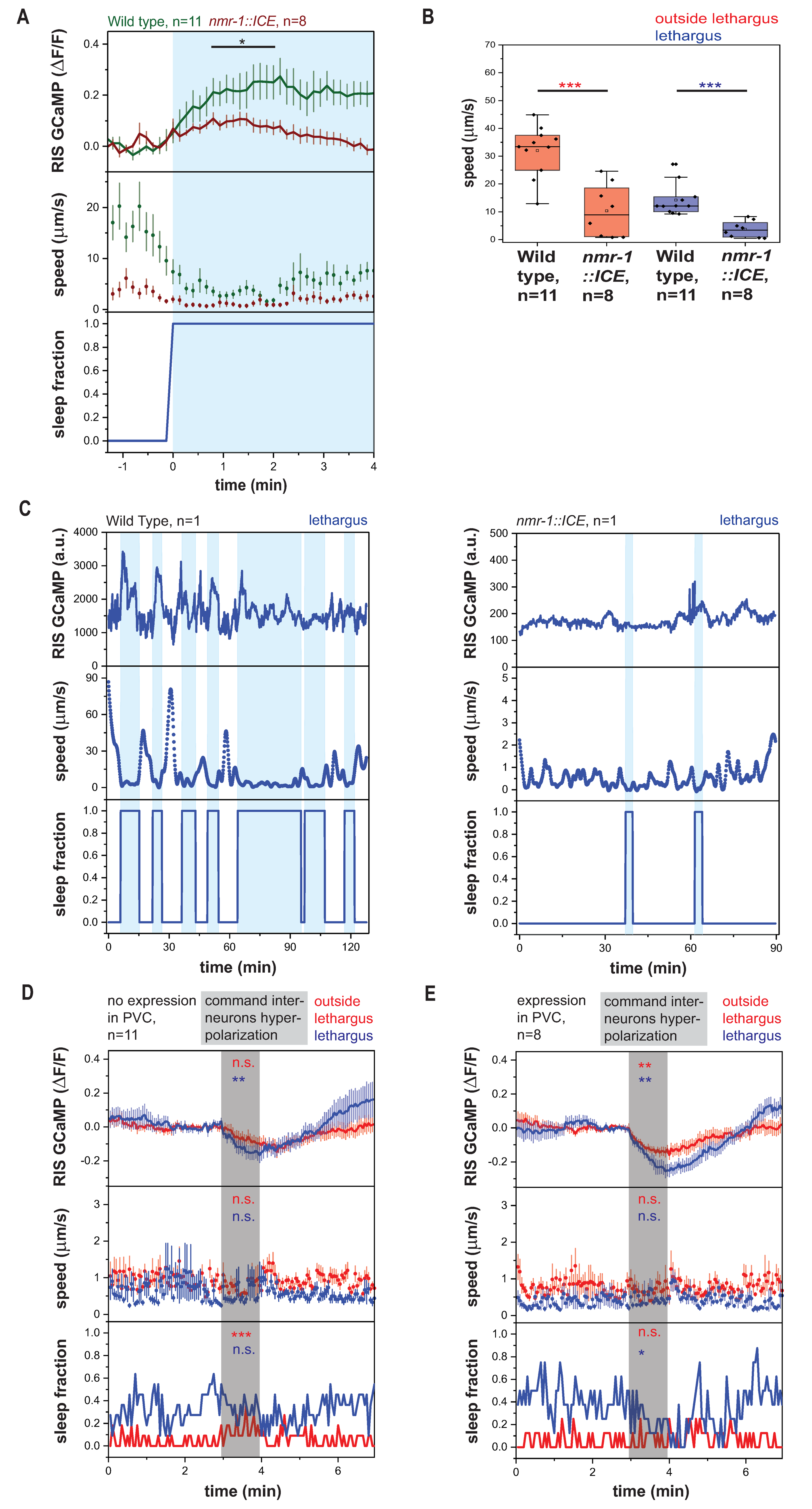

Supplement: S9 Fig — (A) RIS activation in sleep bouts was strongly reduced in command-interneuron–ablated worms. Samples were tested for normal distribution using the Saphiro-Wilk test. *p < 0.05, Welch test (S2 Data, Sheet S9A-C). (B) Command-interneuron–ablated worms moved much slower than wild-type worms. Command interneurons were genetically ablated by expressing ICE from the nmr-1 promoter. Samples were tested for normal distribution using the Saphiro-Wilk test. ***p < 0.001, Welch test for the wake condition and Kolmogorov-Smirnov test for the sleep condition (S2 Data, Sheet S9A-C). (C) Sample traces of RIS activity levels and worm locomotion behaviors outside of and during lethargus in command-interneuron–ablated worms and wild-type worms. In command-interneuron–ablated worms, quiescence bouts occurred only around the middle of the lethargus period (S2 Data, Sheet S9A-C). (D–E) Mosaic analysis of worms expressing an extrachromosomal array of nmr-1::ArchT. Worms were selected that expressed the transgene only in head neurons (D) or head neurons and PVC (E). Neuronal activity levels before (2–2.95 min) and during (3–3.95 min) the stimulation period was compared. *p < 0.05, **p < 0.01, ***p < 0.001, Wilcoxon signed rank test for GCaMP and speed, Fisher’s exact test for sleep fraction (S2 Data, Sheet S9D and S9E). (TIF) [file pbio.3000361.s009.tif]

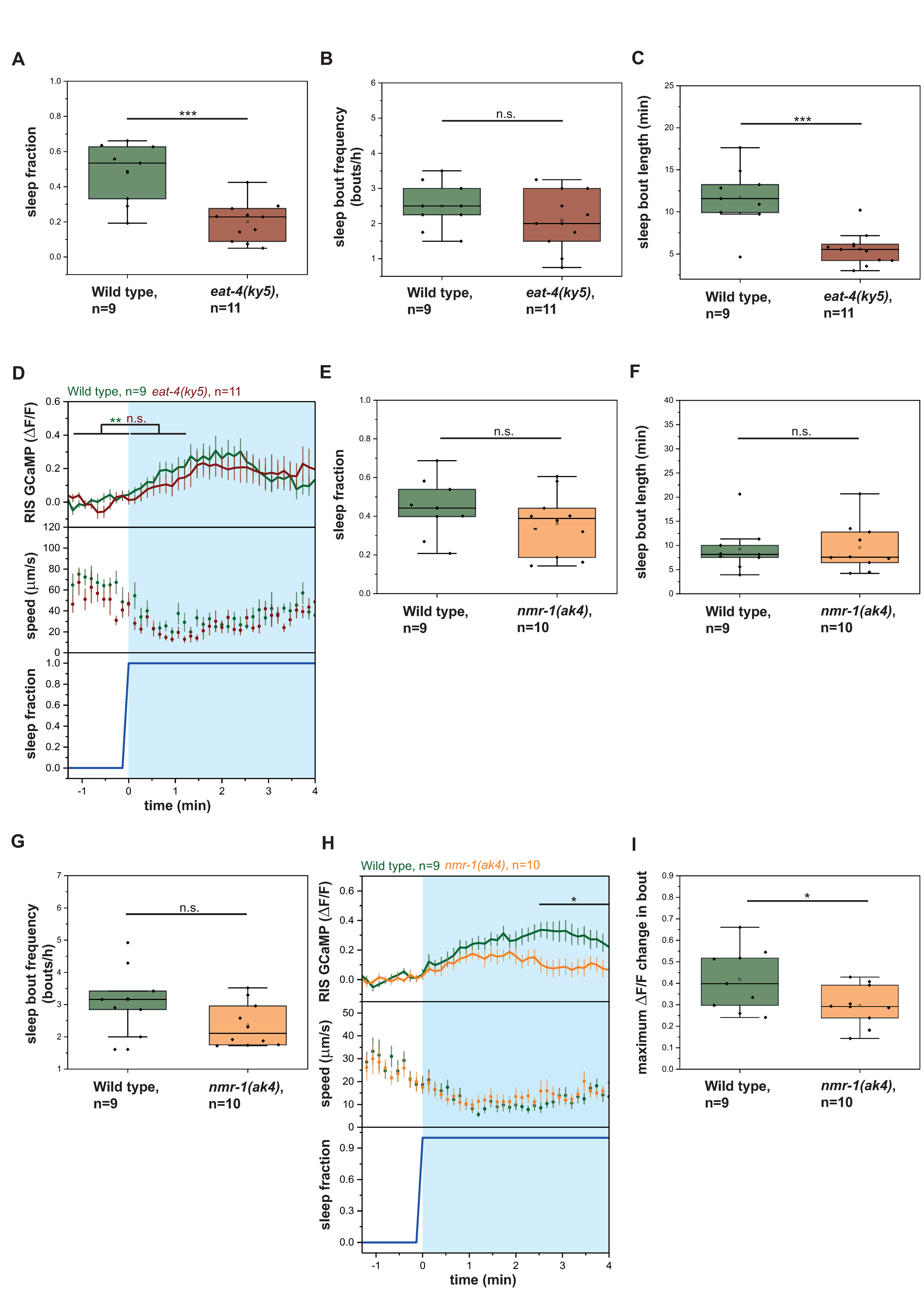

Supplement: S10 Fig — (A–D) Sleep-bout analysis of eat-4(ky5) mutant larvae. eat-4(ky5) animals lacked significant RIS activation at sleep-bout onset. Consistent with this finding, mutant worms displayed a strong reduction in quiescence during lethargus. Samples were tested for a normal distribution using the Saphiro-Wilk test. **p < 0.01, ***p < 0.001, Welch test for comparisons of sleep-bout lengths, sleep-bout frequencies, and sleep fractions. Wilcoxon signed rank test for quantifications of RIS activity levels in sleep bouts (S2 Data, Sheet S10A-D). (E–I) Sleep-bout analysis of nmr-1(ak4) mutant animals. RIS activity levels in sleep bouts were slightly reduced in the mutant. nmr-1(ak4) mutants did not show a reduced amount of quiescence during lethargus. Samples were tested for a normal distribution using the Saphiro-Wilk test. *p < 0.05, Welch test for comparisons of sleep-bout frequencies, sleep fractions, maximum RIS activity levels in sleep bouts, and RIS activity levels at the end of sleep bouts. Kolmogorov-Smirnov test for the comparison of sleep-bout lengths (S2 Data, Sheet S10E-I). (TIF) [file pbio.3000361.s010.tif]

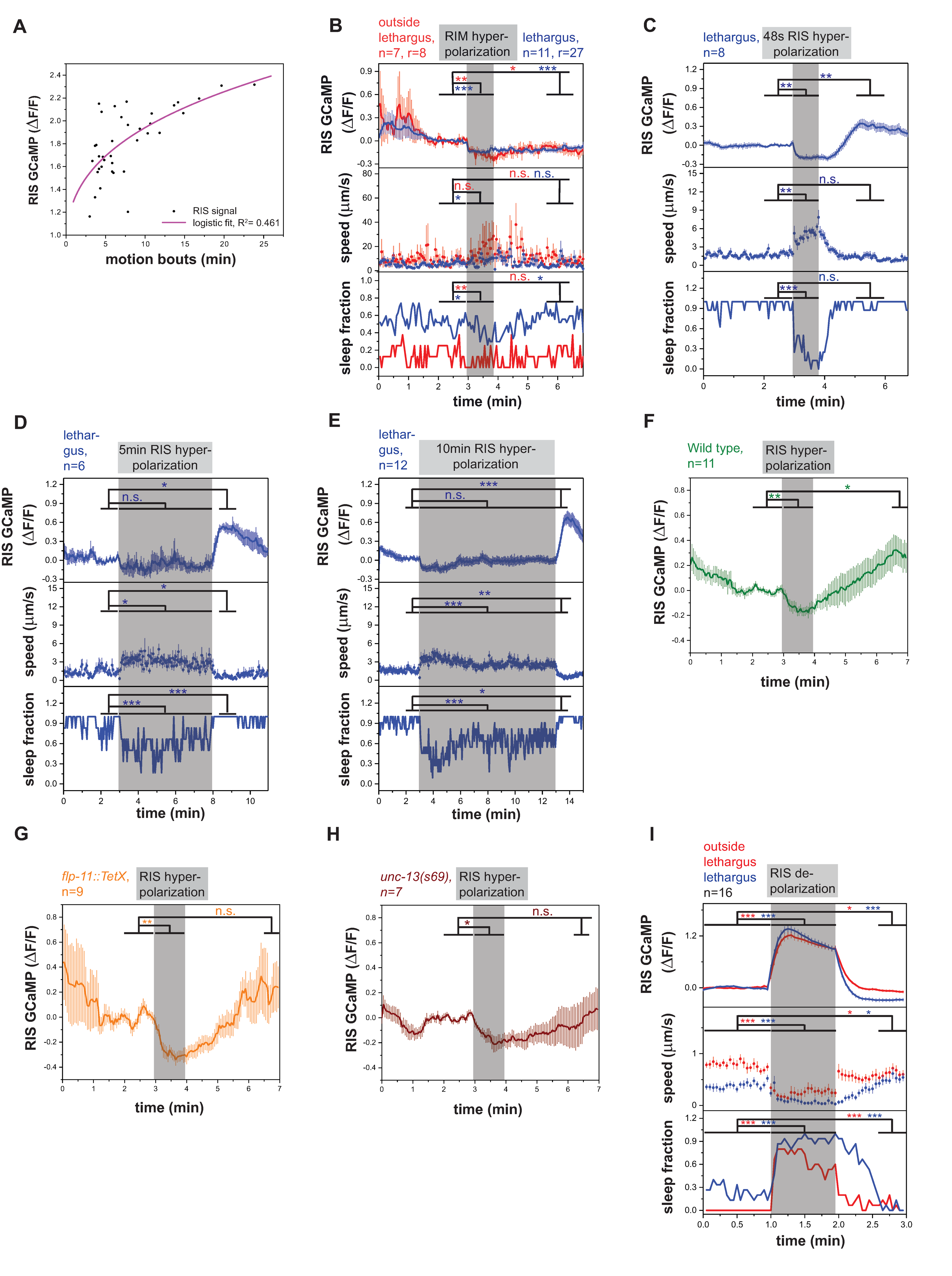

Supplement: S11 Fig — (A) RIS GCaMP transient intensities in wild-type worms are correlated with the length of the preceding motion bout. The longer the preceding motion bout, the stronger the RIS activation (S2 Data, Sheet S11A). (B) RIM was inhibited during and post hyperpolarization. *p < 0.05, **p < 0.01, ***p < 0.001, Wilcoxon signed rank test for GCaMP and speed, Fisher’s exact test for sleep fraction (S2 Data, Sheet S11B). (C) RIS was optogenetically hyperpolarized with stimuli lasting for 48 s (C), 5 min (D), or 10 min (E). Worms not showing a rebound activation transient were excluded from the analysis, which was no worm for 48 s-, 1 out of 7 worms for 5 min-, and 1 out of 13 worms for 10-min stimulation experiments. Data from these plots were used to generate a dose-response curve of optogenetic RIS hyperpolarization (Fig 6D and 6E). *p < 0.05, **p < 0.01, ***p < 0.001, Wilcoxon signed rank test for GCaMP and speed, Fisher’s exact test for sleep fraction (S1 Data, Sheet 6D,E). (F–H) Following optogenetic hyperpolarization, RIS displayed strong rebound activation during lethargus (F). Rebound activation was abolished in a strain that is deficient for neurotransmission specifically in RIS (flp-11::TetX). (G) Rebound activation was abolished also by a mutation that impaired global synaptic transmission (unc-13(s69)). (H) *p < 0.05, **p < 0.01, Wilcoxon signed rank test (S2 Data, Sheet S11F-H). (I) RIS showed a negative rebound following its own optogenetic depolarization. The strength of the negative rebound transient depended on the lethargus status of the worm. Worms during lethargus displayed a 3-times-stronger negative rebound compared to worms outside of lethargus. *p < 0.05, **p < 0.01, ***p < 0.001, Wilcoxon signed rank test for GCaMP and speed, Fisher’s exact test for sleep fraction (S2 Data, Sheet S11I). (TIF) [file pbio.3000361.s011.tif]

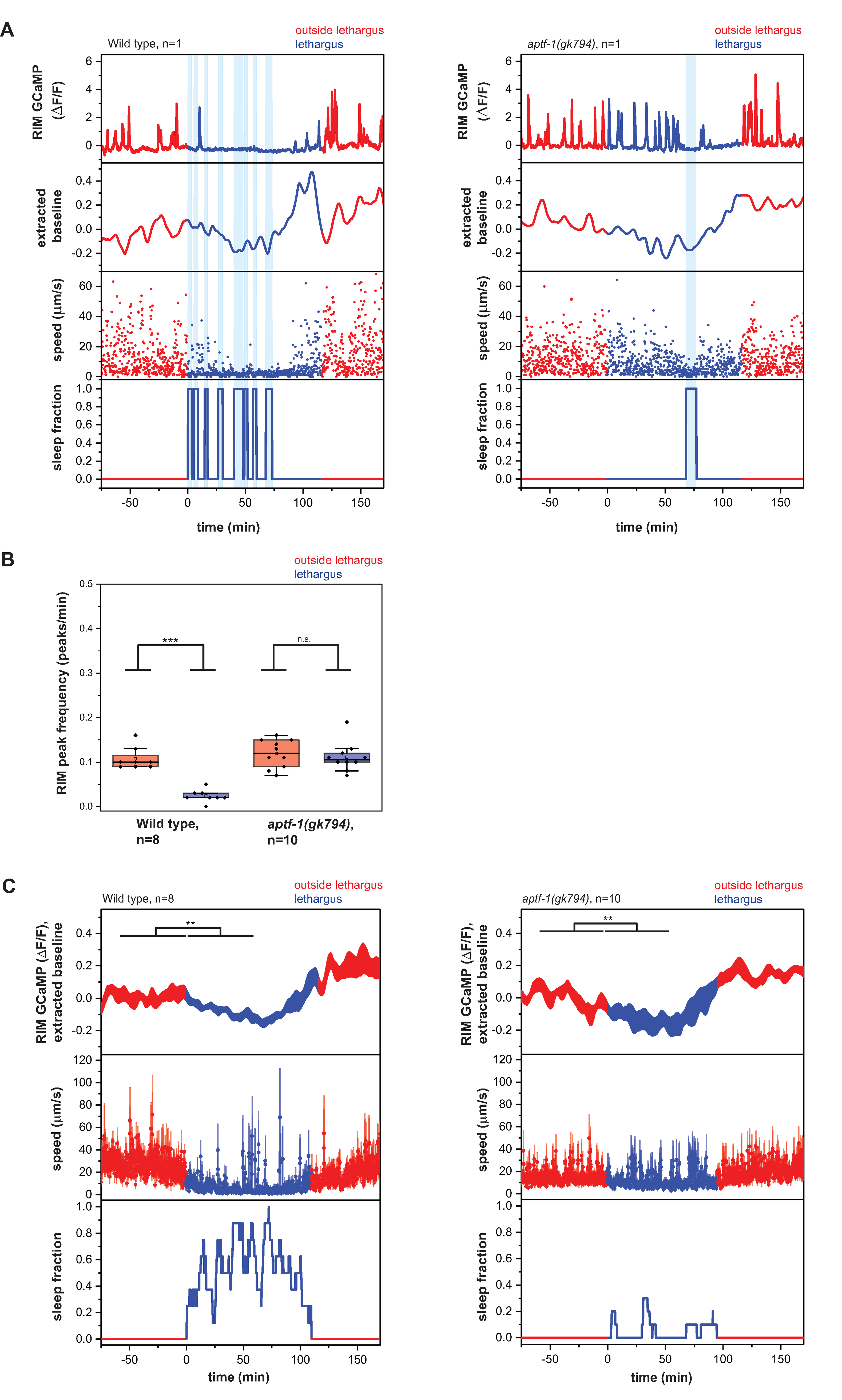

Supplement: S12 Fig — (A) Sample traces of RIM transient frequencies, RIM baseline activities, and worm locomotion behaviors outside of and during lethargus in wild-type worms and aptf-1(gk794) mutants (S2 Data, Sheet S12). (B) Wild-type worms, but not aptf-1(gk794) mutant worms, display changes in RIM transient frequencies across lethargus. Transient frequencies were assessed manually. To be counted as a transient, RIM activity levels had to be at least twice as high as baseline activity levels. ***p < 0.001 Kolmogorov-Smirnov test for wild-type condition, Welch test for mutant condition (S2 Data, Sheet S12). (C) The reduction of RIM baseline activity levels during lethargus is preserved in aptf-1(gk794) mutants. **p < 0.01, Wilcoxon signed rank test (S2 Data, Sheet S12). (TIF) [file pbio.3000361.s012.tif]

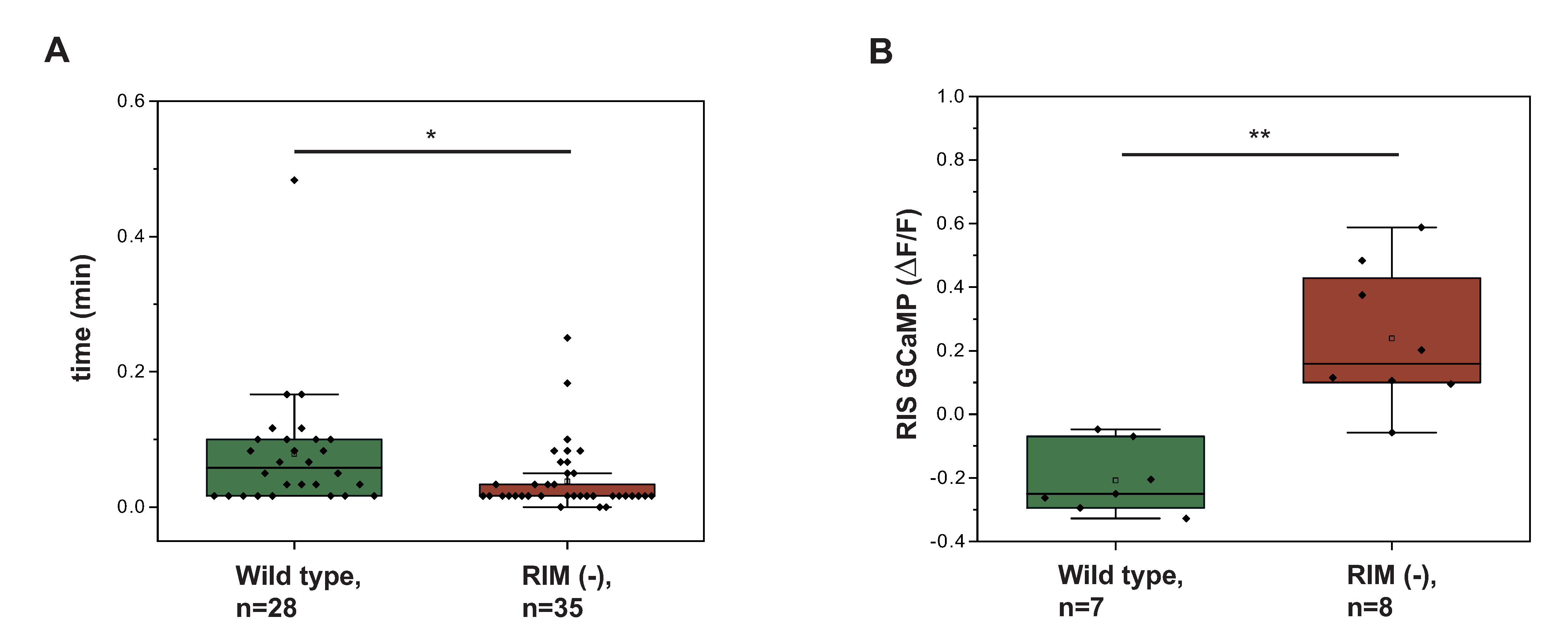

Supplement: S13 Fig — (A) RIM ablation increases the reinstating of immobility following gentle tail touch during lethargus. *p < 0.05, Kolmogorov-Smirnov test (S2 Data, Sheet S13A). (B) RIM ablation increases RIS activation in response to gentle tail touch. **p < 0.01. Kolmogorov-Smirnov test (S2 Data, Sheet S13B). (TIF) [file pbio.3000361.s013.tif]
