# Supplementary material for: A wake-active locomotion circuit depolarizes a sleep-active neuron to switch on sleep
Source: PLoS Biol. 2020 Feb 20;18(2):e3000361. doi: 10.1371/journal.pbio.3000361 (PMC7053779; doi:10.1371/journal.pbio.3000361)
Supplement: S2 Text — (DOCX) [file pbio.3000361.s015.docx]

**S2 Text. List of constructs generated during this study.**

K31 *nmr-1p::SL1-GCaMP3.35-SL2::mKate2-unc-54-3UTR, unc-119(+)*

K78 *tdc-1p::SL1-GCaMP3.35-SL2::mKate2-unc-54-3UTR, unc-119(+)*

K133 *nmr-1p::SL1-GCaMP6s-SL2::mKate2-unc-54-3UTR, unc-119(+)*

K183 *nmr-1p::ReaChR::mKate2-unc-54-3UTR, unc-119(+)*

K189 *tdc-1p::ReaChR::mKate2-unc-54-3UTR, unc-119(+)*

K190 *tdc-1p::ArchT::mKate2 unc-54 3'UTR, unc-119(+)*

K196 *gcy-13p::ArchT::SL2 mKate2 unc-54 3'UTR, unc-119(+)*

K197 *gcy-13p::ReaChR::mKate2-unc-54-3UTR, unc-119(+)*

K200 *nmr-1p::ArchT::SL2 mKate2 unc-54 3'UTR, unc-119(+)*

K204 *tol-1p::ReaChR::mKate2-unc-54-3UTR, unc-119(+)*

K215 *flp11p::ReaChR::SL2mKate2-unc-54-3UTR, unc-119(+)*

K249 *dat-1p::ReaChR::mKate2-unc-54-3UTR, unc-119(+)*

K257 *lad-2p::ReaChR::mKate2-unc-54-3UTR, unc-119(+)*

K259 *dat-1p::ArchT::SL2 mKate2 unc-54 3'UTR, unc-119(+)*

K260 *tol-1p::ArchT::SL2 mKate2 unc-54 3'UTR, unc-119(+)*

K300 *lad-2p::ArchT::SL2 mKate2 unc-54 3'UTR, unc-119(+)*

K308 *tdc-1p::egl-1::SL2mKate2-unc-54-3UTR, unc-119(+)*

K309 *nmr-1p::egl-1::SL2mKate2-unc-54-3UTR, unc-119(+)*

K345 *tbh-1p::ReaChR::mKate2-unc-54-3UTR, unc-119(+)*

*K355 hlh-34p::ReaChR::mKate2-unc-54-3UTR, unc-119(+)*

*K356 hlh-34p::ArchT::SL2 mKate2 unc-54 3'UTR, unc-119(+)*

K364 *zk673.11p::ArchT::SL2 mKate2 unc-54 3'UTR, unc-119(+)*
