# Supplementary material for: A wake-active locomotion circuit depolarizes a sleep-active neuron to switch on sleep
Source: PLoS Biol. 2020 Feb 20;18(2):e3000361. doi: 10.1371/journal.pbio.3000361 (PMC7053779; doi:10.1371/journal.pbio.3000361)
Supplement: S3 Text — (DOCX) [file pbio.3000361.s016.docx]

**S3 Text. Sequence of the strain PHX816.**

**PHX816: *flp-11(syb816 [SL2::mKate2::linker(GSGSG)::tetanustoxin_LC]) X.***

>flp-11b-SL2(gpd-2)-mKate2 linker (GSGSG) tetanustoxin LC
TGGCACTTCTCCTTATTGTCTTCGTTGCCGCTTCTTTTGCTCAATCTTATGATGACGTCAGgtatagttttttcttaaaacaatttttatcaattacccatataaatctattgtagTGCGGAGAAACGTGCCATGCGGAACGCCTTGGTTCGATTTGGAAGAGCTAGTGGTGGAATGAGAAATGCTCTCGTTAGATTCGGAAAGAGGTCTCCATTGGACGAGGAAGACTTTGCTCCAGAGAGCCCACTCCAGGGAAAACGGAACGGTGCCCCACAACCATTTGgtaagttgtcttaaaatttttcttccgctttttgcctttgcttcatgtgtcgtttattttgctttgcagttcgctttggccgatccggtcaactcgaccacatgcacgaccttttgtcgactcttcagAAGCTCAAGTTCGCCAACAACAAGTAATG**ACC**GAGGACGACCGTCTTCTGCTCGAACAACTCCTG**CGA**CGAATTCATCATTAAgctgtctcatcctactttcacctagttaactgcttgtcttaaaatctatgcttctctttagtatctaaaattttcctagaagcttacaagtatataaatggtctcttctcaataaaggttgtatatttattcatcttattgaatctgccatttcctcgtttttgcgagtttatataccttccaattttctttctattgtattttcaacttctaattttaattcagggaaactgcttcaacgcatcATGTCCGAGCTCATCAAGGAGAACATGCACATGAAGCTCTACATGGAGGGAACCGTCAACAACCACCACTTCAAGTGCACCTCCGAGGGAGAGGGAAAGCCATACGAGGGAACCCAAACCATGCGTATCAAGgtaagtttaaacatatatatactaactaaccctgattatttaaattttcagGCCGTCGAGGGAGGACCACTCCCATTCGCCTTCGACATCCTCGCCACCTCCTTCATGTACGGATCCAAGACCTTCATCAACCACACCCAAGGAATCCCAGACTTCTTCAAGCAATCCTTCCCAGAGGGATTCACCTGGGAGCGTGTCACCACCTACGAGGACGGAGGAGTCCTCACCGCCACCCAAGACACCTCCCTCCAAGACGGATGCCTCATCTACAACGTCAAGATCCGTGGAGTCAACTTCCCATCCAACGGACCAGTCATGCAAAAGAAGACCCTCGGATGGGAGGCCTCCACCGAGACCCTCTACCCAGCCGACGGAGGACTCGAGGGACGTGCCGACATGGCCCTCAAGCTCGTCGGAGGAGGACACCTCATCTGCAACCTCAAGgtaagtttaaacatgattttactaactaactaatctgatttaaattttcagACCACCTACCGTTCCAAGAAGCCAGCCAAGAACCTCAAGATGCCAGGAGTCTACTACGTCGACCGTCGTCTCGAGCGTATCAAGGAGGCCGACAAGGAGACCTACGTCGAGCAACACGAGGTCGCCGTCGCCCGTTACTGCGACCTCCCATCCAAGCTCGGACACCGTGGATCCGGATCCGGAATGCCAATCACCATCAACAACTTCCGTTACTCCGACCCAGTCAACAACGACACCATCATCATGATGGAGCCACCATACTGCAAGGGACTCGACATCTACTACAAGGCCTTCAAGATCACCGACCGTATCTGGATCGTCCCAGAGCGTTACGAGTTCGGAACCAAGCCAGAGGACTTCAACCCACCATCCTCCCTCATCGAGGGAGCCTCCGAGTACTACGACCCAAACTACCTCCGTACCGACTCCGACAAGGACCGTTTCCTCCAAACCATGGTCAAGCTCTTCAACCGTATCAAGAACAACGTCGCCGGAGAGGCCCTCCTCGACAAGATCATCAACGCCATCCCATACCTCGGAAACTCCTACTCCCTCCTCGACAAGTTCGACACCAACTCCAACTCCGTCTCCTTCAACCTCCTCGAGCAAGACCCATCCGGAGCCACCACCAAGTCCGCCATGCTCACCAACCTCATCATCTTCGGACCAGGACCAGTCCTCAACAAGAACGAGGTCCGTGGAATCGTCCTCCGTGTCGACAACAAGgtaagtttaaacagttcggtactaactaaccatacatatttaaattttcagAACTACTTCCCATGCCGTGACGGATTCGGATCCATCATGCAAATGGCCTTCTGCCCAGAGTACGTCCCAACCTTCGACAACGTCATCGAGAACATCACCTCCCTCACCATCGGAAAGTCCAAGTACTTCCAAGACCCAGCCCTCCTCCTCATGCACGAGCTCATCCACGTCCTCCACGGACTCTACGGAATGCAAGTCTCCTCCCACGAGATCATCCCATCCAAGCAAGAGATCTACATGCAACACACCTACCCAATCTCCGCCGAGGAGCTCTTCACCTTCGGAGGACAAGACGCCAACCTCATCTCCATCGACATCAAGAACGACCTCTACGAGAAGACCCTCAACGACTACAAGGCCATCGCCAACAAGCTCTCCCAAGTCACCTCCTGCAACGACCCAAACATCGACATCGACTCCTACAAGCAAATCTACCAACAAAAGTACCAATTCGACAAGGACTCCAACGGACAATACATCGTCAACGAGGACAAGTTCCAAATCCTCTACAACTCCATCATGTACGGATTCACCGAGATCGAGCTCGGAAAGAAGTTCAACATCAAGACCCGTCTCTCCTACTTCTCCATGAACCACGACCCAGTCAAGATCCCAAACCTCCTCGACGACACCATCTACAACGACACCGAGGGATTCAACATCGAGTCCAAGGACCTCAAGTCCGAGTACAAGGGACAAAACATGCGTGTCAACACCAACGCCTTCCGTAACGTCGACGGATCCGGACTCGTCTCCAAGCTCATCGGACTCTGCAAGAAGATCATCCCACCAACCAACATCCGTGAGAACCTCTACAACCGTACCGCCTAAaaatcatatgtttttct
