# Supplementary material for: A wake-active locomotion circuit depolarizes a sleep-active neuron to switch on sleep
Source: PLoS Biol. 2020 Feb 20;18(2):e3000361. doi: 10.1371/journal.pbio.3000361 (PMC7053779; doi:10.1371/journal.pbio.3000361)
Supplement: S1 Table — (DOCX) [file pbio.3000361.s017.docx]

**S1 Table. Primers used in this study.**

|  | Primer sequence 5’-3’ |
| --- | --- |
| *aptf-1(gk794)* |  |
|  | CGACAATCTTCCCAAAGACC |
|  | CGGATCGATTGCTAGAGAGG |
|  | GCTTGGACGGCTTTAGTTGA |
| ArchT |  |
|  | ACTTCATCGTCAAGGGATGG |
|  | CATGCAGATGGTGGAGAAGA |
| *eat-4(ky5)* |  |
|  | GGGGCGTTTCCTTTTCTTTA |
|  | AAAATGCTCCGACTCCGATT |
|  | ACAGATCCATACGGAAAAGTTC |
| *flp-11(tm2706)* |  |
|  | CAGGAGTTGTTCGAGCAGAA |
|  | TCGTCCAATGGAGACCTCTT |
|  | TAGCCGCTCGTCTCACTTTT |
| *flp-18(db99)* |  |
|  | CGAACGAATCAGCCATGTAA |
|  | GAGATTCGACGATGACACGA |
|  | GGCTTGGGAGGAAGATTTTT |
| ICE |  |
|  | CCGAGCTTTGATTGACTCCG |
|  | AGTCATGTCCGAAGCAGTGA |
| *nmr-1(ak4)* |  |
|  | TGCTGGTGACTTATGAGCCT |
|  | TGCTGGCGATCTTACTGGAA |
|  | CAACACCGATGCAGAGCTC |
| *tdc-1(n3420)* |  |
|  | \| GAGGATCCACGCCAGAATGA \| \| --- \| |
|  | CATGTGAATCCGCCCAGAAG |
