# Supplementary material for: A wake-active locomotion circuit depolarizes a sleep-active neuron to switch on sleep
Source: PLoS Biol. 2020 Feb 20;18(2):e3000361. doi: 10.1371/journal.pbio.3000361 (PMC7053779; doi:10.1371/journal.pbio.3000361)
Supplement: Attachment — Submitted filename: point-by-point-response_20191115.pdf [file pbio.3000361.s022.pdf]

Point-by-point response to reviewer comments

Reviewer #1:

I think this manuscript is a well conducted study that employs a series of circuit manipulations that nicely demonstrate the role of the PVC neuron in activating the sleep active RIS neuron in C. elegans.
The authors give a thorough introduction to the question they are trying to elucidate. Here, this reviewer really believes that the use of such a powerful model as C.elegans with its stereotype and complete connectome is of great benefit to the question at hand. This gives a lot of credit to the study.
This reviewer believes that the conclusions made by the authors are supported by the data and more importantly that the paper manages to provide a reasonably clear explanation of the complex interactions occurring between the different types of neurons assayed in this study.
This reviewer has no major concerns regarding this manuscript and believes it could be publish as it is.

Authors:
We would like to thank the reviewer for the positive feedback.

Reviewer #2:

A key outstanding question in understanding sleep/wake control is “how is behavioral state stabilized?”, i.e. “Why is there no rapid switching between sleep and wake states?” as would be expected from an electronic flip-flop switch. One proposal is that there is an excitatory input (e.g. orexin neuropeptides in mammals) to wake-active neurons, which stabilizes their activity. Here, Maluk et al propose an alternative (or additional) explanation for behavioral state stability. Based on their circuit-based analysis of sleep in C. elegans, they propose that a pair of neurons (called PVC) that is involved in wake behavior, also forms reciprocal excitatory connections with a sleep inducing neuron (called RIS) to create a reinforcing positive feedback loop to stabilize the sleep state. This model is particularly provocative since it invites a reconsideration of a view held for over 35 years that the main role of PVC neurons is to promote forward locomotion in response to mechanical
stimulation of the posterior part of the body. The authors additionally suggest a circuit-based mechanism for the homeostatic regulation of sleep.

While the results are solid and support their model, there are a few key experiments missing that would shore up the key claims.

1. The claim that PVC activation is part of the circuit for sleep initiation is supported by their neuronal activation and silencing experiments. However, these optogenetic manipulations are artificial and could possibly generate non-physiological responses. If their model is correct, one would expect to see spontaneous PVC activity increase coincident with RIS activity and during lethargus quiescent bouts.

Authors:
As suggested by the reviewer, we tested whether RIS and PVC calcium transients coincide in lethargus. We imaged calcium activity simultaneously in both RIS and PVC. We used a custom-written MATLAB script to extract all RIS peaks, averaged these and aligned PVC activity to the RIS peaks. This analysis showed that PVC started activating 2 minutes before RIS peaks and stayed active when RIS activity is initially rising (Figure 2A). This data is consistent with the idea that PVC is an activator of RIS. Similar results were obtained for RIM (Figure 4A).

2. The authors show that experimental depolarization of RIS leads to a rebound reduced calcium activity whereas hypopolarization of RIS leads to rebound increased calcium activity. However, they did not show whether such apparent homeostatic regulation is unique to RIS or whether it also occurs in PVC or neurons. This is important since neuronal activity and synaptic homeostasis have been widely described in both mammals and invertebrates, even in neurons not involved in sleep regulation. Moreover, an experiment that could distinguish between homeostatic mechanisms intrinsic to RIS and a circuit based mechanism is to repeat these experiments in a genetic background in which synaptic transmission is blocked (e.g. using unc-13 mutants).

Authors:
Depolarization rebound (negative rebound):
We tested for a negative rebound in PVC neurons. After PVC depolarization, we also saw a robust rebound activity decrease outside of and in lethargus (Figure S7A). Thus, at least PVC responds to optogenetic depolarization in a similar manner as RIS. The rebound activity decreases after optogenetic

depolarization could thus be a general effect and might not be specific to RIS. However, both RIS and PVC act in circuits controlling sleep and locomotion, and it is possible that such an effect is typical for neurons in this circuit. We observed that the negative rebound of RIS showed a state dependency as it was still stronger during lethargus (Figure S11I), whereas such state dependency was not visible in PVC. This suggests that this experiment reveals lethargus-specific regulation of RIS. Because from these results we could not clearly show that the negative rebound is not a general effect of neurons we de-emphasized these data and moved them into the supplement. We focus the story now on the positive rebound, for which be obtained clear evidence that it is an RIS-specific effect (see below)

Hyperpolarization rebound (positive rebound):
We started by testing the effects of optogenetic PVC inhibition on PVC activity. Optogenetic inhibition of PVC did not cause any rebound activation following the stimulation period neither outside nor in lethargus (Figure 2C). In addition, we performed this experiment with RIM. As in the PVC experiment, we could not see a rebound activation transient following the stimulation period outside of and in lethargus (Figure S11B). This indicates that the rebound following optogenetic inhibition is a specific property of RIS. We added the optogenetic inhibition to the main manuscript figure (PVC) or to the supplement (RIM) and refer to these experiments as demonstrations of the specificity of the positive rebound effect in RIS.

These experiments also showed that outside of lethargus, both RIM and PVC remained inhibited after the optogenetic stimulus. RIM could also be inhibited during lethargus. Interestingly, PVC became resistant to inhibition specifically during lethargus. This suggests that the capacity of PVC to be inhibited is decreased during lethargus, and we hypothesize that this effect contributes to the shift in locomotor interneuron circuit activity that is required for increasing sleep during lethargus. We have hence included the PVC inhibition experiments to the main manuscript and discuss these results.

Synaptic vs. cell-intrinsic mechanisms:
As suggested, we repeated the optogenetic hyperpolarization of RIS in *unc-13(s69)* mutant animals (Figure S11H). Additionally, we performed the same experiments in worms, which expressed tetanus toxin specifically in RIS, a condition that abolished neurotransmission specifically in RIS (Figure S11G). In both conditions, RIS did not show significant rebound activation after hyperpolarization. Thus, we conclude that the RIS rebound activation transient is mediated by an extrinsic, circuit-based mechanism. We added these data as supplementary figures and mention these results in the main text.

3. Lines 499-501. The authors proposal that the degree of PVC activation determines whether it causes forward locomotion or activates RIS to cause quiescence is testable. It would predict that weak PVC optogenetic stimulation results in forward movement whereas strong stimulation results in quiescence. This would seems to be a simple experiment for them to do with existing reagents. It would allow them to potentially reconcile the apparently conflicting functions of PVC.

Authors:
We performed this experiment as suggested. We used expression of ReaChR from the nmr-1 promoter. To ensure PVC-specific optogenetic stimulation the tail was illuminated specifically. Specific PVC activation always led to forward motion (Figure 2D). This experiment is consistent with the idea that PVC is an important activator of RIS, but it also suggested that PVC (optogenetic) activation alone is not sufficient for strong RIS activation that is required for sleep induction.

In search for additional RIS activators we reinvestigated the role of the RIM neurons. This analysis showed that inhibition of the RIMs reduces RIS activity (Figure 1C, RIM panel), supporting the view that RIM is an additional activator of RIS. Like PVC, however, RIM does not seem to be sufficient for strong RIS activation that is necessary for sleep bout induction (Figure 4B-E). Simultaneous inhibition of multiple interneurons that are known to control locomotion showed strong inhibition of RIS and loss of sleep (Figure 5), suggesting that multiple neurons, including RIM and PVC, are required for RIS activation during sleep.

We therefore refined our model. Because we found a role in sleep induction for a forward and a reverse command interneuron, we developed the idea that sleep induction might happen at the transition from forward to reverse locomotion states, at a time where both PVC and RIM have overlapping activities.

The optogenetic effect of PVC and RIM activation on behavior are shown in the main manuscript (Figure 2D, 4E). Additional data on the concerted action of multiple locomotion neurons was added, and the model was refined. The present model reconciles the known role of PVC in forward movement with its role in inducing sleep. It links sleep control to locomotion control, and provides a model for how sleep states are switched (Figure 9).

I noted several issues with the text that need corrections. These writing issues and other minor points are listed below.

1. Last sentence in abstract “A tripartite flip-flop switch likely also underlies sleep control in mammals.” They provide no literature support to this claim. I suggest instead changing “…likely also underlies…” to “…may also underlie…”

Authors:
We changed this accordingly.

2. In the abstract, the authors say “PVC is inhibited by reverse command interneurons, which are stimulated by arousing cues.” Yet in their bullet Highlight points you say “Wakefulness-activated PVC forward command interneurons”. It is not clear to the reader what the difference is between “arousing cues” and “wakefulness”.

Authors:
We tried to emphasize that neurons, which are known to be active during wakefulness, can act as RIS activators. We edited the paper to make this clearer.

3. Highlight point “RIS activity is homeostatically regulated in both directions” is unclear. I would suggest revising to “RIS activity shows compensatory homeostatic regulation following its depolarization or hyperpolarization”.

Authors:
We removed Highlight points to comply with journal style.

4. Last highlight point. It is unclear what the authors mean by “neuronal dampening”. Do they mean “developmental time dependent global dampening of neural activity”?

Authors:
We removed Highlight points to comply with journal style.

5. The authors use the term “tiredness” several times but I think they mean “sleepiness”. “Tiredness” is less specific as it can also refer to muscle fatigue and to mental fatigue that does not reflect sleep/wake regulation.

Authors:
As recommended, the term tiredness was exchanged by the term sleepiness.

6. Lines 48-49: “Sleep deprivation is detrimental to human health and is associated with an increased risk of infection, cardiovascular disease, depression, obesity, type 2 diabetes, and cancer”. The authors should be careful about these claims (also they provide no references for this sentence.). A more accurate capture of the known literature would “Short sleep is associated with an increased risk of infection (REFs), cardiovascular disease (REF), depression (REF), obesity (REF), type 2 diabetes (REF), and cancer (REF)”.

Authors:
The above-mentioned sentence was changed in the main text according to the reviewer’s recommendation.

7. Lines 52-56 on aplysia and STG ganglia are irrelevant to this paper. Remove.

Authors:
The lines about Aplysia and STG ganglia were removed from the main text.

8. Lines 61: unclear what they mean by the sentence “The depolarization of sleep-active neurons defines the key properties of sleep behavior”.

Authors:
The above-mentioned text section was rephrased to improve its readability.

9. At some point, the authors need to state that the PVC neurons are a pair (not a single neuron).

Authors:
This information was added to the main text.

10. Lines 124-125, unclear what "reverse arousal" means. Do they mean "backwards command interneurons and thus inhibit..." ?

Authors:
This text passage was rephrased.

11. Can the variability in the RIM activation effects on RIS be explained by timing of stimulation (early vs late lethargus)?

Authors:
To answer that question lethargus periods of animals tested in former Figure 1B and 1C (now Figure S1D) were split into 3 equally sized sections: lethargus onset, middle of lethargus and end of lethargus. In each interval, the amount of trials with an RIS activation, RIS inhibition and no change in the RIS signal were normalized to the total number of trials in that interval. They suggest no dependency of RIM effects on the timing in lethargus (Figure S1E). Instead we found that different neurotransmitters in RIM are responsible for the different effects on RIS. RIM can inhibit RIS via tyramine and FLP-18 and can activate RIS through glutamate (Figure S2, S3). We added this information to the manuscript.

12. The authors use the terms “optogenetic gain-of-function” and “optogenetic loss of function”. These are terms used to describe gene variant effects but are confusing when used to refer to optogenetic neuronal activation and silencing experiments.

Authors:
The phrase “optogenetic gain-of-function” experiments was replaced by optogenetic depolarization experiments in the main text. The term “optogenetic loss-of-function” experiments was replaced by optogenetic hyperpolarization experiments.

13. The authors show that command interneuron ablation results in reduced sleep but the more important experiment directly relevant to their model is to ablate PVC specifically. It appears that they have a selective promoter for doing such a kill genetically (perhaps combining miniSOG expressed under their PVC+ promoter coupled with exposure of the tail to blue light).

Authors:
We tried to ablate PVC using miniSOG, which was expressed under the *nmr-1* promoter. For this, we specifically illuminated the tail with blue light. However, we had problems ablating PVC, because the miniSOG protocol is unfortunately not yet established for the application in L1 larvae. Two independent researchers in our lab tried to ablate PVC using miniSOG. One of the main problems was that L1 larvae died because of the amount of light, which was needed to ensure PVC ablation.

To achieve a more specific PVC loss-of-function condition, we performed experiments using the *zk637.11* promotor, which has no overlap with the expression pattern of the *nmr-1* promoter except for PVC. In *zk637.11::ArchT* experiments performed in worms in lethargus we could show that inhibition of PVC inhibits sleep (Figure 2B).

14. Figure S4 should be discussed in greater detail. It appears that eat-4 (S4E) is required for sleep maintenance (bout duration) but not for sleep onset (bout frequency). This should be mentioned. Also, nmr-1 is minimally required (S4A) suggesting that other glutamate receptors play a role here.

Authors:
We agree to the reviewer’s possible explanation of the *eat-4(ky5)* and *nmr-1(ak4)* mutant phenotypes and according to the reviewer’s suggestion we have commented on that in our main text.

15. Lines 216-220 sentence is long and unclear: “While PVC has previously been shown to promote forward locomotion[45], its inhibition leads to increased mobility implying that the reduction of locomotion after simultaneous ablation of most command interneurons stems from the reverse command interneurons that promote mobility, whereas PVC appears to play a predominant role in dampening motion behaviors through activation of RIS.”

Authors:
The above-mentioned sentence was restructured and rephrased.

16. Lines 268-271: “These results are consistent with the idea that sleep induction is a self-enforcing process in which RIS-mediated inhibition of brain activity promotes further RIS activation (Figure 2F).” An alternative explanation for the observations of aptf-1 mutants is that, in this mutant, the RIS neuron is intrinsically defective in more ways than just lacking flp-11. Doing this experiment in flp-11 mutants would be cleaner and more convincing.

Authors:
We repeated the experiment with *flp-11(tm2706)* mutant animals and included these additional results in the figure. The results, like those from *aptf-1(gk794)*, showed defects in inducing long RIS calcium transients (Figure 3H-J). We added this information to the main figures.

17. Lines 34 4-345: “Hyperactive mutants suppress sleep across species including C. elegans [49-51]”. The authors should cite should also cite Nagy et al ELife 2013 and Belfer et al, Sleep 2013 for C. elegans hyper mutants and Cirelli et al Nature 05, Koh et al Science 08, and Kume et al J. Neurosci 05 for Drosophila hyper mutants suppressing sleep.

Authors:
We added the additional references.

18. Line 388: Give reference for tdc-1 promoter.

Authors:
We provided a reference for the *tdc-1* promoter. For a detailed description of the *tdc-1* promoter as well as all other promoters and plasmids used in this study please check the material and methods section (Table S2).

19. Line 512. I’m not aware of recordings from humans. Do they mean rodents? In this and many other sentences, they do not include a literature reference.

Authors:
We rewrote this section and added citations.

20. Methods line 584: indicate which of the strains listed were generated during the course of these experiments. For those strains previously described, give reference.

Authors:
Strains generated for this study are now indicated. Strains that we were provided with and which were described previously are also indicated and references are provided. This information can be found in the material and methods section in the paragraph about worm maintenance and strains.

21. Figure 1CD. Why does RIS activity remain low outside lethargus after PVC inhibition? Similar result in 1D. This bears comment.

Authors:
We optogenetically inhibited PVC and simultaneously measured PVC activity (Figure 2C). The result of this experiment showed that after optogenetic PVC inhibition in wake worms, PVC activity levels stayed significantly below baseline for a time interval of 3 minutes following the stimulation period. However, in worms in lethargus PVC activity did not reduce substantially. This resistance of PVC to inhibition and the sustained inhibition of PVC outside of lethargus could explain the effects on RIS activity. We added this experiment to the main text. This new finding is exciting as it suggests that the resistance of PVC to inhibition specifically during lethargus is an important feature of the locomotion circuit that favors RIS activation during lethargus.

22. Figure 1C: How do you know that inhibition was successful in neurons for which the Arch experiments showed no effect on RIS?

Authors:
All optogenetic tools we were using were fused to an mKate2 fluorescent protein. mKate2 fluorescence was used to confirm that optogenetic tools were indeed expressed in neurons of interest. The genetic tool (ArchT) that we used for inhibition was the same for all conditions. However, it is still possible that the method does not work efficiently in some of the neurons. We have added the information on how we confirmed tool expression and discuss the possibility that the method may not have worked in some neurons. The method was successful to discover key roles for PVC and RIM neurons and this led to a

model that can explain sleep state switching. Perhaps, there are additional circuit components to regulate RIS and these, if they exist, can be studied in the future with improved tools.

23. Line 895: It was not clear to me why SDQL data was treated differently statistically.

Authors:
SDQL data was treated differently, because RIS baseline activity levels were constantly dropping before the stimulation period up to the timepoint of 0.6 minutes in lethargus. Only from that time point onwards baseline activity levels were stabilizing and considered as statistically relevant to be compared to RIS activity levels during the stimulation period. To keep data analysis consistent, we also took a shorter baseline for conditions outside of lethargus. A comment concerning the statistical analysis was added to the figure caption.

--

Reviewer #3:

Maluck et al describe a sleep-regulatory circuit in C. elegans that resembles the flip-flop architecture that has been observed in mammals. The authors combine optogenetic manipulation and GCaMP imaging in behaving worms to map the connectivity of sleep regulatory neurons during and outside lethargus. The manuscript is well organized and begins to outline the organization of sleep-influencing neurons upstream of RIS, but the following points should be addressed to strengthen the paper:

1) The roles of PVC in directing locomotion and sleep remain unclear. The authors should more clearly describe how PVC activity might promote both locomotion and sleep. Could PVC have two different modes of activity? Does varying the intensity of optogenetic RIS stimulation have different effects on sleep/locomotion?

Authors:
We agree that the role of PVC in promoting both locomotion and sleep was unclear. We performed additional experiments and refined our model:

Different intensities of light for PVC stimulation did not induce a different behavior but PVC activation always led to forward locomotion (Figure 2D). We therefore revised and improved our model and provide evidence that while PVC is a strong RIS activator, its activation is not sufficient to induce sleep.

Reanalysis of the RIM neuron showed that RIM is an important activator of RIS in addition to PVC (Figure 1B, C). The strongest sleep loss phenotype was observed in a condition that ablated or impaired both PVC and RIM (as well as other command interneurons) (Figure 5). PVC and RIM are both active before sleep bouts but are normally activated during locomotion states that are known to oppose each other. This suggests that overlapping activity from PVC and RIM might occur at the transition between forward and reverse locomotion states in lethargus, causing strong RIS activation and sleep. This refined model can explain how locomotion interneurons can activate a sleep-active neuron as well as promote locomotion.

2) PVC activation increases RIS calcium levels during lethargus, but has no effect or slightly decreases calcium in RIS outside of lethargus. It is not clear how this might occur – does lethargus alter neuromodulatory levels that might render RIS sensitive to PVC activity? Does PVC excitability change during lethargus?

Authors:
We thank the reviewer for these interesting questions. To test for a possible change in the excitability of PVC, we optogenetically activated PVC using ReaChR and simultaneously measured its activity before, during and after the optogenetic stimulation (Figure S7A). These experiments were performed with worms being outside of and in lethargus. We found that optogenetic PVC depolarization led to the same amount of PVC activity increase during stimulation periods outside of and in lethargus. From that we concluded that PVC excitability remains unchanged in lethargus.

Interestingly though, when we hyperpolarized PVC and simultaneously measured PVC GCaMP we could see that the inhibitory levels of PVC differed outside of and in lethargus (Figure 2C). During the inhibition, the activation levels of PVC dropped more outside of lethargus compared to during lethargus. Furthermore, after the stimulation, PVC activity went back to baseline levels only during lethargus. Outside of lethargus, PVC stayed at a reduced activity level. We conclude that not the excitability but the inhibition of PVC is state dependent.

This result suggests that the resistance to inhibition of PVC during lethargus is an important step in shifting the equilibrium of the locomotion interneurons towards RIS activation. We added these experiments to the manuscript and discuss these findings.

3) While PVC activation increases GCaMP signal in RIS during lethargus but not outside lethargus, PVC hyperpolarization reduces GCaMP in RIS both during and outside lethargus. The authors should discuss how to reconcile these results.

Authors:
To answer this question, we optogenetically inhibited PVC and simultaneously measured PVC activity (Figure 2C). The result of this experiment showed that after optogenetic PVC inhibition in worms outside of lethargus, PVC activity levels dropped significantly and stayed significantly below baseline for a time interval of 3 minutes following the stimulation period. However, in worms in lethargus PVC activity was not reduced substantially. By contrast, optogenetic activation of PVC caused the same levels of activation both during and outside of lethargus (Figure S7A).

This resistance of PVC to inhibition and the sustained inhibition of PVC outside of lethargus could explain the different effects of depolarization and hyperpolarization on RIS activity. We added the PVC hyperpolarization experiment to the main text and the PVC depolarization experiment to the supplement. These results are exciting as they suggest that the resistance of PVC to inhibition specifically during lethargus is an important feature of the locomotion circuit that favors RIS activation during lethargus.

4) If PVC is activated by RIS, then would PVC responses to RIS activation be ablated in aptf-1 mutants that lack neuropeptidergic output from RIS neurons?

Authors:
For specific RIS activation we expressed ReaChR under the *flp-11* promoter. APTF-1 is a transcription factor required for expression of the sleep-inducing neuropeptide FLP-11, hence the optogenetic tool is not functional in *aptf-1* deletion mutants. However, we conducted this experiment in *flp-11(tm2706)* mutants, which are more specific in ablating neuropeptidergic function of RIS (Figure 3C). We tested PVC's response to RIS activation in *flp-11(tm2706)* mutants and this showed that the PVC activation is almost abolished in the absence of neuropeptide signaling from RIS. We added this experiment to the main manuscript figures.

5) Lines 240-243 – It is not clearly described how a sleep-command neuron would promote sleep by being active during both sleep and wake.

Authors:
We refined our model. Our new data showed that PVC and RIS activity do not precisely coincide, but that PVC (and also RIM) activates prior to RIS (Figure 2A,4A). Our refined model suggests that RIS is activated at the transition from forward to reverse locomotion states, at times when both PVC and reverse interneurons such as RIM have overlapping activity on RIS. We describe the refined model in our revised manuscript and describe PVC to be active not during sleep but at its onset (Figure 9).

6) Is the rebound excitation observed in RIS neurons after optogenetic inhibition unique? Do other neurons also show similar homeostatic excitability after acute inhibition? Similarly, is RIS activation increased following mechanical stimulation during lethargus that delays sleep?

Authors:
According to the reviewer's suggestions we optogenetically inhibited PVC and RIM neurons and simultaneously measured activity levels in these neurons (Figure 2C, S11B). After optogenetic PVC or RIM inhibition we could not detect a significant rebound activation transient, neither outside of nor in lethargus. From that we concluded that the rebound activation transient, which occurred after optogenetic RIS inhibition, is unique to RIS.

Following the recommendations of reviewer 2, we repeated the RIS optogenetic inhibition experiment in *unc-13*(s69) mutants as well as worms expressing tetanus toxin, specifically in RIS (Figure S11F-H). In both strains, the rebound could no longer be observed. This shows, that the RIS rebound after optogenetic inhibition is dependent on synaptic signaling to RIS.

To answer the second question, we performed mechanical stimulation experiments in worms, which were outside of and in lethargus and simultaneously measured RIS activity levels (Figure S7B). Per trial

worms were tapped in total 2 times, each with a 1 second break in between the taps. Both outside of and in lethargus mechanical stimulation caused a strong RIS activity increase. Also, both outside of and in lethargus worms clearly mobilized during mechanical stimulation. However, in lethargus worms strongly immobilized shortly after the mechanical stimulation and mobility levels even dropped below baseline levels. Contrary to that outside of lethargus worms displayed speed levels following mechanical stimulation, which were comparable to those before the mechanical stimulation.

These data support a specific role of rebound activation in RIS and the responsiveness of RIS to mechanical stimulation. We added these data to the main manuscript and to the supplement.

7) The model posed in Figure 6 proposes that sleep regulatory signals are relayed through AVE and AVA, but they are not examined in this manuscript. If AVE and AVA act as the wake active arm of a flip-flop switch, then their activity should be suppressed during lethargus. Would AVE and AVA be redundant, or would silencing one be sufficient to suppress arousal and promote sleep? These questions seem central to the flip-flop model.

Authors:
We agree that the wake promoting sleep inhibiting arm of the circuit could involve AVE and AVA neurons. It has been shown before that the activities of AVE and AVA are suppressed during lethargus. Turek et al. 2013 showed in their Figure1J that the transient frequency per minute in AVE/AVA is significantly reduced in lethargus in wildtype worms compared to the wake state. This is not the case in non-sleeping *aptf-1(gk794)* mutants.

In order to understand which neurons of AVE and AVA have the arousing function, we tested worms with ablated AVE or AVA neurons (Figure 3G). AVE ablation led to a significant increase of sleep during lethargus, whereas the ablation of AVA did not. These experiments pointed to a prominent role of AVE in mediating the inhibition action of RIS. We hence tested PVC activity upon RIS depolarization in worms with ablated AVE neurons (Figure 3D-F). This experiment showed that the activation is significantly reduced in lethargus, which confirms AVE to be an important arousal player in the homeostatic circuit. We added these data to the main manuscript.

--

Reviewer #4:

In this manuscript, Maluck et al. investigate the network regulating the activity of the RIS neuron. This neuron plays a key role in C. elegans developmentally timed sleep, and thus understanding how its activity is regulated is crucial for the elucidation of sleep in this organism and of the general principles of sleep/wake regulation. Using mainly calcium imaging and optogenetics, they show that the PVC neuron is required for RIS activation during sleep, and that RIS can also upregulate PVC activity. Interestingly, they find that none of the pre-synaptic partners of RIS are inhibitory. Based on these observations they suggest that a positive feedback loop upregulate the activity in both neurons during sleep and act as a flip flop mechanism. They also show that continuous inhibition or activation of RIS is followed by compensatory responses both in terms of neuronal activity and in terms of sleep/wake regulation, suggesting that such responses could be involved in sleep homeostasis on a short time scale. Finally, they provide evidence that awakening by a stimulation of the ASH neuron requires the RIM neuron, which is downstream of ASH and upstream of RIS as well as of AVA, a command inhibitory neuron upstream of PVC. I would have two major comments:

1) The authors provide strong evidence that PVC is necessary and sufficient to activate RIS during sleep. They also show that optogenetic stimulation of RIS activates PVC, raising the idea of a positive feed-back loop between the two neurons. Showing that RIS inhibition reduces PVC activity would provide strong support in favor of this feed-back loop, however experiments inhibiting RIS activity are difficult to interpret because such manipulations simultaneously induce wakefulness. Thus, the idea that a positive feed-back loop takes place between the two neurons is mainly based on RIS artificial stimulation and data from the literature. The authors suggest in particular that inhibitory inputs linked to reverse locomotion and arousal are likely a key element of this feed-back loop, because they can be modulated by RIS. Ablating specifically the inhibitory input neuron directly connected to both RIS and PVC, i.e. AVE, stimulating RIS and recording PVC activity would provide experimental evidence for this hypothesis, but is not included in the manuscript.

Authors:
We agree that testing PVC's response to RIS depolarization in AVE ablated worms is an important experiment. If the feedback was indirect through inhibition of AVE, it should be reduced in AVE ablated

worms. This was the case. The activation of PVC in AVE-ablated worms is clearly reduced compared to wildtype worms (Figure 3D-F).

We wanted to go a step further and investigate how RIS inhibits AVE and therefore activates PVC indirectly. We hence tested if the sleep-inducing and RIS specific neuropeptide FLP-11 was responsible for this feedback loop. In fact, the activation of PVC upon RIS depolarization is almost completely abolished in *flp-11(tm2706)* mutants (Figure 3C). The feedback loop is therefore FLP-11-dependent and PVC gets activated indirectly by RIS through inhibition of PVC inhibitors with FLP-11.

The discussion brings forward a flip-flop mechanism based on command interneurons, however other models are possible. Experiments shown in Figure 1 show that the inhibition of PVC does not provide a full awakening and indicate that besides PVC, other neurons with direct synaptic connection to RIS could modulate its activity. It remain possible that manipulating individually each of the non-PVC neurons is insufficient to affect RIS but that a more coordinated inhibition – or activation - of those neurons could tip the balance towards sleep or wakefulness.

Authors:
We agree that it is possible that additional neurons may be important regulators of RIS and that concerted action of multiple neurons likely is required for the full level of complexity of RIS regulation.

We have addressed this comment by further analyzing neurons that are presynaptic to RIS and have found a role for RIM in activating RIS which led to a refinement of the model for RIS activation during sleep.

1) To test whether a coordinated inhibition of all RIS upstream neurons could induce RIS activation, we tried to generate a line, which expresses ArchT in all presynaptic neurons. Unfortunately, worms which were positive for the ArchT transgenes were not viable. For that reason, we decided to limit the amount of ArchT transgenes expressed in upstream neurons to the minimum amount of two. We decided to pair ArchT expression in the CEP and URY neurons for the following reasons:

1) For PVC and RIM we found that their function is interconnected in order to induce RIS activation. See below.
2) SDQL is most probably not yet connected to RIS in the L1 larvae.
3) CEP and URY neurons are both classified as sensory neurons.

Simultaneous optogenetic inhibition of CEP and URY neurons did not induce any changes in RIS activity levels and we added this information to the supplement (Figure S5D).

2) Re-analysis of RIM with a stronger promoter showed that this neuron can act as an activator of RIS (Figure 1C). We found that neither RIM nor PVC optogenetic activation is sufficient for sleep induction (Figure 2D,4E), that concerted inhibition or ablation of both PVC and RIM caused substantial sleep phenotypes (Figure 5), and that RIM and PVC are both active before RIS during sleep (Figure 2A,4A). Hence, we refined our model for RIS activation. Strong activation of RIS for sleep induction may require concerted activation of RIS by RIM and PVC. Such a condition may occur at the transition from forward to reverse locomotion states.

Thus, while it is possible that additional neurons are required for RIS control, we defined a locomotion interneuron circuit for RIS activation during sleep that can explain how sleep states are switched.

2) The evidence that RIS depolarization is under homeostatic control is based on optogenetic manipulation. It is unclear whether the rebounds observed are a specific feature of RIS or could occur in any neurons manipulated the same way- would stimulation of PVC or RIM be also followed by a rebound? These complementary experiments should be included to strengthen the conclusion. Secondly the expression of channel rhodopsin may affect the activity of the neuron in a way that is not representative of the normal physiology. Regarding that issue, the data shown in Figure S7 is particularly relevant, since RIS activation is not optogenetically induced. However it is not clear in Figure S7 what is the difference between “lethargus non mobilizing” and “lethargus mobilizing”: The first condition induces a ΔF/F rebound and reduced sleep after blue light, while the second is showing an opposite pattern of responses. The authors should clarify. Finally, in Figure 3C and S6 the worms not showing rebound activation transient after RIS were excluded from the analysis. The authors should provide information on the % of responding animals; this is important information regarding the robustness of the phenomenon.

Authors:

1) specificity of the activation rebound of RIS:

Depolarization rebound (negative rebound):

We tested for a negative rebound in PVC neurons. After PVC depolarization, we also saw a robust rebound activity decrease outside of and in lethargus (Figure S7A). Consequently, at least PVC responds to optogenetic depolarization in a similar manner as RIS. The rebound activity decreases after optogenetic depolarization could thus be a general effect and might not be specific to RIS. However, both RIS and PVC act in circuits controlling sleep and locomotion, and it is possible that such an effect is typical for neurons in this circuit. We observed that the negative rebound of RIS showed a state dependency as it was still stronger during lethargus (Figure S11I), whereas such state dependency was not visible in PVC. This suggests that this experiment reveals lethargus-specific regulation of RIS. Because from these results we could not clearly show that the negative rebound is not a general effect of neurons we de-emphasized these data and moved them into the supplement. We focus the story now on the positive rebound, for which we obtained clear evidence that it is an RIS-specific effect (see below)

Hyperpolarization rebound (positive rebound):

We started by testing the effects of optogenetic PVC inhibition on PVC activity (Figure 2C). Optogenetic inhibition of PVC did not cause any rebound activation following the stimulation period neither outside nor in lethargus. In addition, we performed this experiment with RIM (Figure S11B). As in the PVC experiment, we could not see a rebound activation transient following the stimulation period outside of and in lethargus. This indicates that the rebound following optogenetic inhibition is a specific property of RIS. We added the optogenetic inhibition to the main manuscript figure (PVC) or to the supplement (RIM) and refer to these experiments as demonstrations of the specificity of the positive rebound effect in RIS.

These experiments also showed that outside of lethargus, both RIM and PVC remained inhibited after the optogenetic stimulus. RIM could also be inhibited during lethargus. Interestingly, PVC became resistant to inhibition specifically during lethargus. This suggests that the capacity of PVC to be inhibited is decreased during lethargus, and we hypothesize that this effect contributes to the shift in locomotor interneuron circuit activity that is required for increasing sleep during lethargus. We have hence included the PVC inhibition experiments to the main manuscript and discuss these results.

2) blue light experiment:

We included more information about the blue light experiment in the main text, which we hope can explain the difference between “lethargus non-mobilizing” and “lethargus mobilizing” in more detail.

3) data exclusion:

Furthermore, additional information was added to the figure captions of former figures 3B, now Figure 6D-E (see main text lines) and former figure S6, now S11C-E (see supplementary information lines) to indicate how many worms were excluded from the analysis because they did not show a rebound activation transient. In case of 48 seconds optogenetic stimulation all worms displayed a rebound activation transient (0% were excluded). In case of 5 minutes stimulation 1 out of 7 worms was excluded (14%) and in case of 10 minutes stimulation 1 out of 13 worms was excluded (8%).

Other comments:
-Figure 1: Please comment on the fact that RIS is not activated in all sleep episodes, for example episodes 6 and 7 in panel A.

Authors:
We thank the reviewer for that very interesting observation. We think that in episode 6 there is a noticeable local increase of RIS activity correlating with the detected sleep bout. In case of episode 7 it might be that the sleep bout, which was initiate by the RIS activity increase was to short to be detected by our algorithm. Sleep bouts were defined as periods of no movement of at least 2 minutes. It might be the case that the worm was immobile for less than 2 minutes. Additionally, our circuit model would predict that the less a worm is active during a motion bout in lethargus the smaller the following RIS transients should be, and this is now stated in the discussion.

-Figure 3B: The ΔF/F data shown in the right panel does not seem to match with what is shown in figure 3C.

Authors:
The data shown in former Figure 3B (now 6D-E) and former Figure 3C (now 11I) stems from different experiments. The data in Figure 3B was generated from experiments, in which RIS was optogenetically inhibited over different time intervals. Individual plots for the different stimulation period lengths are shown in the Figure S11C-E. However, the data depicted in Figure 3C (now 11I) represents the optogenetic depolarization of RIS for the defined stimulation length of 1 minute. We changed the description.

-in Figure 3C, the amount of sleep detected is much lower than in the other experiments. Please provide explanations in the legend.

Authors:
The different amounts of sleeping worms in Figure 3A and Figure 3C were caused by the usage of different thresholds to define sleep. In Figure 3A a worm was considered to be asleep if it moved slower than 30% of its average wake speed. In Figure 3C a worm was considered to be asleep, if it moved slower than 10% of its average wake speed. However, we corrected this parameter in Figure 3C and also used a 30% threshold. The results can now be found in Figure 6C and S11I.

-Methods: there is insufficient detail on how pan-neuronal or command interneuron activity was recorded and analyzed.

Authors:
Additional information on how pan-neuronal and command interneuron activities were measured was added to the material and methods section.

-Methods: how was the specificity of the genetic ablations checked?

Authors:
We co-expressed a fluorescent reporter in the neurons that were targeted for ablation and confirmed with fluorescence microscopy that neuronal integrity was compromised by the expression of apoptosis inducers. Information about the generation of genetic ablations was added to the paragraph about the strain generation in the material and methods part.
