# Supplementary material for: A wake-active locomotion circuit depolarizes a sleep-active neuron to switch on sleep
Source: PLoS Biol. 2020 Feb 20;18(2):e3000361. doi: 10.1371/journal.pbio.3000361 (PMC7053779; doi:10.1371/journal.pbio.3000361)
Supplement: Attachment — Submitted filename: point by point02.docx [file pbio.3000361.s023.docx]

REVIEWS:

Authors’ responses are indicated in blue

Reviewer #2 (David Raizen): 
The authors have addressed all issues I raised. Nice work. 

Authors:

We thank David Raizen for the positive statement and for the comments that have helped us to improve our manuscript.

Reviewer #3: 
The authors have added a considerable amount of new data for this revision and have addressed my previous comments. I have no new critiques to raise.

Authors:

We thank Reviewer 3 for the positive statement and for the comments that have helped us to improve our manuscript.

Reviewer #4: 
The authors have thoroughly addressed the reviewer's requests. The new results reach more nuanced conclusions, while still providing important information regarding sleep regulation. I would have a few remaining comments:

Authors:

We thank Reviewer 4 for the positive statement and for the comments that have helped us to improve our manuscript. We reply to the remaining comments below.

-The data presented in Figure 2A shows the correlation between PVC and RIS transients. Showing the activity of PVC with respect to sleep bout onset, as presented for RIM in Figure 4A, would add equally important information regarding the link between PVC and sleep, as well as providing a point of comparison with RIM activity.

Authors:

We agree that this experiment would be useful to even better compare PVC and RIM activity. We had already tried this experiment before the last revision. The challenge has been that PVC calcium transients are extremely small compared with those of other neurons such as RIM. PVC has been the focus of locomotion control for more than 30 years and has been studied by many labs, and in all these decades nobody has ever managed to measure spontaneous calcium transients in mobile worms. In fact, this report is the first to measure spontaneous PVC transients. This was only possible because we immobilized the worms, which greatly reduced measurement noise. The signal change that we observed in immobilized animals was in the range of 10%. The noise fluctuations in mobile animals was in the range of 20-30%. Thus, all our attempts to measure spontaneous PVC calcium transients in mobile worms and to correlate them with sleep bouts were unsuccessful. The immobilization that was necessary for this measurement precludes the assessment of behavioral quiescence as a readout. However, RIS activity has been shown by us and other labs to correlate precisely with sleep bouts. This allows us to use RIS activity as a proxy for sleep bouts.

We prefer to not include measurements of PVC in mobile animals into the manuscript, which is already quite full, as these measurements only show noise. We have rewritten the results paragraph. We clearly state that PVC cannot be measured in mobile animals and that we use RIS activity as a proxy for sleep bouts.

-Methods: please provide more information on how was the pan-neuronal GCaMP activity evaluated and which neurons were assessed.

Authors:

We added this information to the materials and methods section.

-Methods: please provide more information on how the time threshold of 2 min was chosen for identifying sleep (p22 line 625).

Authors:

We added this information to the materials and methods section.

-Main text, p19, line 573: please correct sentence "… RIM continued showing RIS transients during lethargus… "

Authors:

We have corrected this sentence.
